# Amaryllidaceae Alkaloids of Belladine-Type from *Narcissus pseudonarcissus* cv. Carlton as New Selective Inhibitors of Butyrylcholinesterase

**DOI:** 10.3390/biom10050800

**Published:** 2020-05-22

**Authors:** Abdullah Al Mamun, Jana Maříková, Daniela Hulcová, Jiří Janoušek, Marcela Šafratová, Lucie Nováková, Tomáš Kučera, Martina Hrabinová, Jiří Kuneš, Jan Korábečný, Lucie Cahlíková

**Affiliations:** 1 ADINACO Research Group, Department of Pharmaceutical Botany, Faculty of Pharmacy, Charles University, Heyrovského 1203, 500 05 Hradec Králové, Czech Republic; almamuna@faf.cuni.cz (A.A.M.); hulcovd@faf.cuni.cz (D.H.); janousj2@faf.cuni.cz (J.J.); safratom@faf.cuni.cz (M.Š.); 2 Department of Organic and Bioorganic Chemistry, Faculty of Pharmacy, Charles University, Heyrovského 1203, 500 05 Hradec Králové, Czech Republic; marikoj2@faf.cuni.cz (J.M.); kunes@faf.cuni.cz (J.K.); 3 Department of Pharmacognosy, Faculty of Pharmacy, Charles University, Heyrovského 1203, 500 05 Hradec Králové, Czech Republic; 4 Department of Analytical Chemistry, Faculty of Pharmacy, Charles University, Heyrovského 1203, 500 05 Hradec Králové, Czech Republic; novakoval@faf.cuni.cz; 5 Department of Military Medical Service Organisation and Management, Faculty of Military Health Sciences, University of Defence, Třebešská 1575, 500 05 Hradec Králové, Czech Republic; kucera-t@email.cz; 6 Department of Toxicoloxy and Military Pharmacy, Faculty of Military Health Sciences, University of Defence, Třebešská 1575, 500 05 Hradec Králové, Czech Republic; hrabinova@pmfhk.cz; 7 Biomedical Research Centre, University Hospital Hradec Kralove, Sokolska 581, 500 05 Hradec Kralove, Czech Republic

**Keywords:** Amaryllidaceae, *Narcissus pseudonarcissus* cv. Carlton, alkaloids, carltonine A–C, Alzheimer’s disease, butyrylcholinesterase, docking studies

## Abstract

Thirteen known (**1**–**12** and **16**) and three previously undescribed Amaryllidaceae alkaloids of belladine structural type, named carltonine A-C (**13**–**15**), were isolated from bulbs of *Narcissus pseudonarcissus* cv. Carlton (Amaryllidaceae) by standard chromatographic methods. Compounds isolated in sufficient amounts, and not tested previously, were evaluated for their in vitro acetylcholinesterase (AChE; E.C. 3.1.1.7), butyrylcholinesterase (BuChE; E.C. 3.1.1.8) and prolyl oligopeptidase (POP; E.C. 3.4.21.26) inhibition activities. Significant human BuChE (*h*BUChE) inhibitory activity was demonstrated by newly described alkaloids carltonine A (**13**) and carltonine B (**14**) with IC_50_ values of 913 ± 20 nM and 31 ± 1 nM, respectively. Both compounds displayed a selective inhibition pattern for *h*BuChE with an outstanding selectivity profile over AChE inhibition, higher than 100. The in vitro data were further supported by in silico studies of the active alkaloids **13** and **14** in the active site of *h*BuChE.

## 1. Introduction

Alzheimer’s disease (AD), a progressive neurodegenerative brain disorder featuring memory loss and cognitive impairments, has been named after German psychiatrist Alois Alzheimer. AD is the most common type of dementia among the elderly, generally diagnosed in individuals over the age of 65 years [1]. Considering population aging and increased life expectancy, the number of AD patients is predicted to increase enormously. About 4.6 million new cases are diagnosed every year throughout the world, and the World Health Organization (WHO) estimates that by 2050 over 131 million patients will suffer from AD [2]. Additionally, the current cost of dementia is about a trillion US dollars per year, and it should rise to 2 trillion US dollars by 2030 [3]. Thus, AD starts to be one of the greatest public health problems with severe impact on patients, their families and care workers.

Although the exact pathogenesis of AD remains elusive, it is currently conceived as a multifactorial disease. The main pathological hallmarks of AD are the deposits of β-amyloid peptide (Aβ) in senile plaques and neurofibrillary tangles (NFTs) formed by hyperphosphorylated τ-protein [4]. In addition to them, inflammation and oxidative stress processes also extensively contribute to the loss of synaptic integrity and neurodegeneration.

The current treatment is only symptomatic and mainly involves restoring of acetylcholine (ACh) levels through AChE inhibition [5]. Three AChE inhibitors, namely donepezil, galanthamine and rivastigmine, are currently used as the main therapeutic option for AD treatment [6]. BuChE is another serine hydrolase homologous to AChE that is encoded by a different gene and can be found ubiquitously throughout the body [6]. The role of BuChE has recently gained enormous interest, and not only in the field of AD research [7]. Regarding AD, it is well-documented that AChE activity is downregulated by up to 33–45% of normal values, while the activity of BuChE is escalated by up to 40–90% [8]. This dramatic switch between the AChE/BuChE ratio highlighted the supportive role of BuChE in hydrolysing the excess of ACh. Moreover, several lines of evidence also indicate that both cholinesterases play an important role in Aβ aggregation and maturation to oligomers, fibrils and plaques [9].

POP, a cytosolic serine peptidase, has gained interest as a target for the treatment of neuropsychiatric and neurodegenerative diseases like schizophrenia, bipolar affective disorder, Parkinson’s disease and AD [10]. POP is expressed in several brain regions, and catalyses with high specificity the cleavage of short peptides containing proline at its carboxyl site [11]. POP is responsible for the degradation of neurotransmitters, such as substance P, arginine vasopressin–oxytocin, and neurotensin, which in turn reduce the possibility of generation of toxic Aβ peptides. Generally, POP can be regarded as one of the active players in the processes of learning and memory. In fact, some POP inhibitors have been experimentally found to act as antidementia drugs [10].

Natural products represent an important source of clinical drugs, especially for their structural diversity and wide range of biological activities [12]. Alkaloids are, without a doubt, the most intriguing templates of natural origin [13]. The family Amaryllidaceae comprises about 1100 species in 85 genera, which are distributed worldwide through the tropics and warm regions [14]. Some of the species are cultivated as ornamental plants for their beautiful flowers and fragrant essences. Different types of plant extracts from this family have been long used in traditional medicine for the treatment of cancer by the ancient Greeks, as well as by Africans, Asians, and Polynesian communities for the treatment of various diseases [15,16]. The medicinal value of the Amaryllidaceae species is attributed to the presence of tyrosine-derived alkaloids, typically named as Amaryllidaceae alkaloids (AAs), that are produced exclusively by this family. These alkaloids exhibit a wide range of biological activities, including AChE inhibition, antitumor, antiviral, antimalarial, analgesic antibacterial, and cytotoxic properties. One successful example of an Amaryllidaceae alkaloid is exemplified by galanthamine, a drug indicated for the treatment of mild to moderate vascular dementia and AD [17].

*Narcissus* L. is the most common genus of the Amaryllidaceae family comprising 80–100 wild species, mainly distributed in Southwestern Europe and North Africa, with some populations in the Balkans, Italy and France. Plants of the genus *Narcissus* L. have been used in traditional medicine worldwide in cancer therapy [18,19,20,21]. Most of the species can hybridize, thus, a large number of cultivars have been developed for ornamental purposes. Interestingly, some intersectional hybrids and cultivars have also been reported as potential sources of galanthamine and other Amaryllidaceae alkaloids [22,23]. The cultivar Carlton is cultivated for the commercial extraction of galanthamine, because of its relatively high concentration in the bulbs, the large bulb size, and their availability in large volumes. Galanthamine is reported as the major alkaloid of *Narcissus pseudonarcissus*, followed by haemanthamine. Haemanthamine also has interesting biological activities including inhibition of protein synthesis, antimalarial and antiretroviral activity, as well as cytotoxicity against various cancer cell lines [24,25,26]. Recently, some semi-synthetic derivatives of haemanthamine have been published as promising candidates for AD therapy [27].

As a part of our ongoing research on Amaryllidaceae alkaloids with implication to AD, this work reports the isolation of several such alkaloids from fresh bulbs of *Narcissus pseudonarcissus* cv. Carlton. The isolated alkaloids that had not been previously studied were submitted for biological evaluation to reveal their inhibition potential towards *h*AChE, *h*BuChE, and POP. In vitro data were further supported by investigating the compound’s putative binding modes in the active site of *h*BuChE to display crucial interaction.

## 2. Results and Discussion

### 2.1. Phytochemical Study of Narcissus pseudonarcissus cv. Carlton

**Figure 1:**
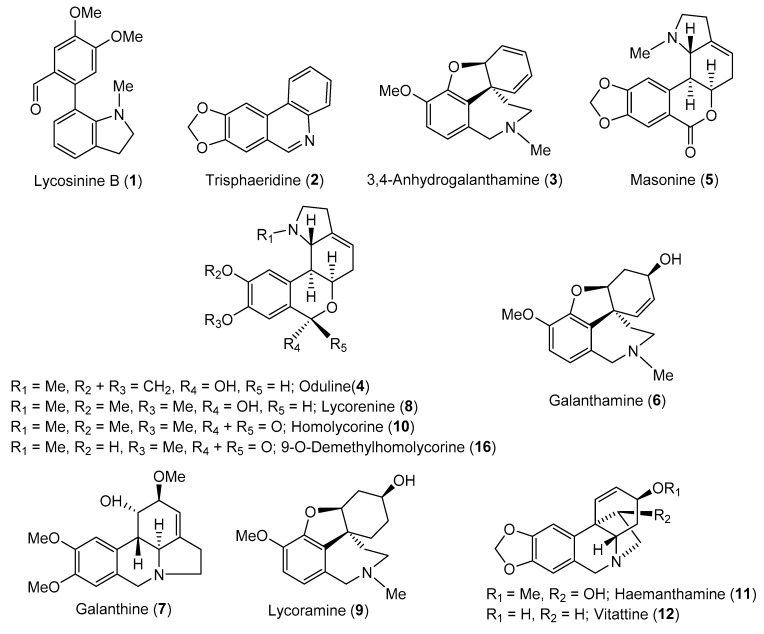
Structures of isolated alkaloids from *Narcissus pseudonarcissus* cv. Carlton.

Thirteen known (**1**–**12** and **16**) and three novel AAs (**13**–**15**) were isolated from bulbs of *Narcissus pseudonarcissus* cv. Carlton (Amaryllidaceae) by common chromatographic methods, as described in the Experimental section. The compounds were identified by MS, ESI-HRMS, 1D and 2D NMR spectroscopic analyses and by comparison of the obtained data with the literature (Figure 1). These techniques led to the identification of lycosinine B (**1**) [28], trisphaeridine (**2**) [29], 3,4-anhydrogalanthamine (**3**) [30], oduline (**4**) [31], masonine (**5**) [32], galanthamine (**6**) [33], galanthine (**7**) [34], lycorenine (**8**) [33], lycoramine (**9**) [33], homolycorine (**10**) [31], haemanthamine (**11**) [20], vittatine (**12**) [35], and 9-*O*-demethylhomolycorine (**16**) [31]. The isolated alkaloids belong to the galanthindole (**1**), narciclasine (**2**), galanthamine (**3, 6, 9**), homolycorine (**4, 5, 8, 10, 16**), lycorine (**7**) and haemanthamine (**11, 12**) structural types; newly isolated alkaloids **13**–**15** belong to the beladinne-type of AAs.

**Figure 2:**
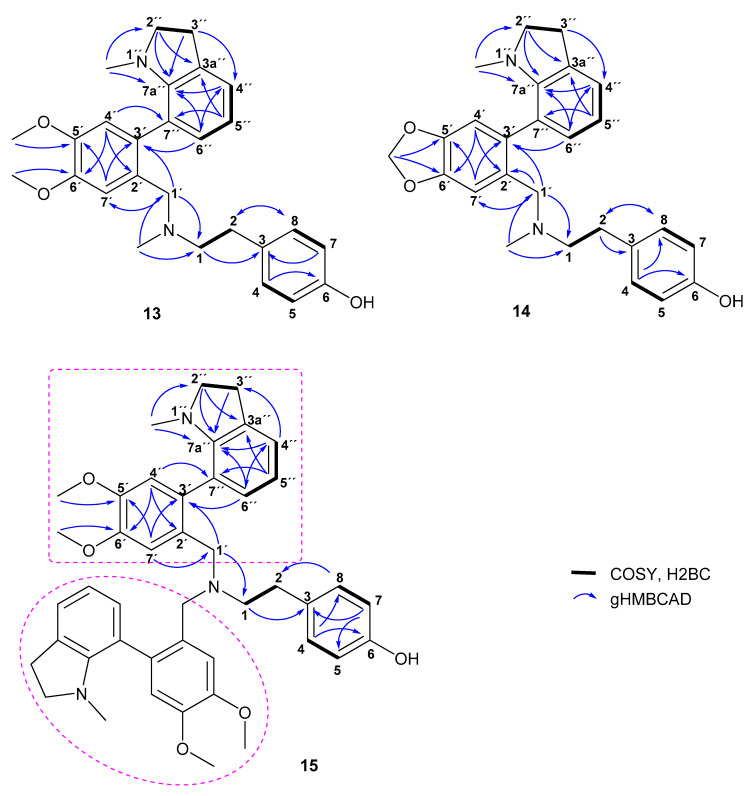
Key 2D NMR correlations of compounds **13**–**15**.

The new compound **13,** named carltonine A, was obtained as a pale yellow oil. The HRMS of **13** showed a protonated molecular ion peak [M + H]^+^ at *m/z* 433.2488, corresponding to the molecular formula C_27_H_32_N_2_O_3_ (433.2486 calcd for C_27_H_33_N_2_O_3_^+^). The ^1^H NMR spectrum exhibited resonances associated with nine aromatic protons from which the presence of a *p*-substituted benzene ring (*δ*_H_ 7.00–6.96; 6.72–6.68), a 1,2,4,5-tetrasubstituted benzene ring (*δ*_H_ 7.10, 6.76) and a 1,2,3-trisubstituted benzene ring (*δ*_H_ 7.09, 6.82, 6.72) were obvious. The high-field part of the ^1^H NMR spectrum contained one singlet corresponding to two methoxy groups (*δ*_H_ 3.34), two doublets (*δ*_H_ 3.45, 3.29), two doublets of triplets (*δ*_H_ 3.32, dt, overlap, *J* = 17.3, 8.8 Hz; 3.20, dt, *J* = 17.3, 8.8 Hz), two triplets (*δ*_H_ 2.98, t, *J* = 8.8 Hz; 2.65, t, *J* = 7.4 Hz) and two singlets of *N*-methyl groups (*δ*_H_ 2.21, 2.20). The ^13^C and HSQC data revealed signals of four deshielded sp^2^ carbons (*δ*_C_ 154.0, 150.4, 148.1, 146.9), five nonprotonated sp^2^ carbons (*δ*_C_ 132.5, 132.1, 131.2, 130.1, 123.0), seven aromatic methines (*δ*_C_ 130.5, 129.8 (2C), 123.3, 117.9, 115.1 (2C), 113.2, 111.1), three deshielded methylenes (*δ*_C_ 59.2, 58.5, 57.1), two methoxy groups (*δ*_C_ 55.9, 55.8), two *N*-methyls (*δ*_C_ 42.3, 38.6) and two aliphatic methylene groups (*δ*_C_ 32.8, 28.6). The COSY spectrum, supported by H2BC correlations, showed cross-peaks for the H-1/H-2, H-2″/H-3″, H-4″/H-5″/H-6″ and AA′BB′ spin systems. Moreover, these assignments corresponded to the spin-spin splitting of signals in the ^1^H spectrum. HMBC correlation from H-2 to the sp^2^ carbons at *δ*_C_ 129.8 revealed the fact that the *p-*substituted benzene ring was attached to the aminoethyl group. The nitrogen from this substructural fragment bore a methyl at *δ*_C_ 42.3 and a benzylic methylene at *δ*_C_ 58.5. The 1,2,4,5-tetrasubstituted aromatic ring was determined by HMBC correlations from H-4′ and H-7′. This experiment also revealed the conjunction of this fragment with the methylene C-1′ by C-2′. The HMBC cross-peaks of *δ*_H_ 6.76 to *δ*_C_ 123.0 and *δ*_H_ 6.82 to *δ*_C_ 132.1 indicated the attachment of nonprotonated carbons C-3′ and C-7″. This sp^2^ quaternary carbon belonged to the remaining 1,2,3-trisubsituted aromatic ring which was a part of the *N*-methylindoline moiety. This was proven by correlation of its protons to the related carbons. Therefore, the structure of **13** was established as depicted (Figure 2). The assigned atoms are shown in Table 1.

Carltonine B (**14**) was obtained as a pale yellow oil. The molecular formula was determined to be C_26_H_28_N_2_O_3_ from the protonated molecular ion peak [M + H]^+^ found at *m*/*z* 417.2172 (417.2173 calcd for C_26_H_29_N_2_O_3_^+^) in the positive-ion HRMS. Due to the small quantity of sample, the ^1^H NMR spectrum had poor resolution, but it looked similar to that of **13**. In addition, the ^13^C spectrum was in accordance with this observation (see Table 1). The only difference was the absence of signals assigned to two methoxy groups of the 1,2,4,5-tetrasubstituted aromatic fragment, which were replaced by signals corresponding to a strongly deshielded methylene group of a 1,3-dioxole moiety in the 1D spectra. The key correlations are presented in Figure 2.

Another new compound, carltonine C (**15**), was obtained as a yellowish amorphous solid. The 1D NMR spectrum, as well as the 2D NMR spectra of 15 and 13, were quite similar; however, the ^13^C NMR data showed doubling of most carbon resonances and the absence of the *N*-methyl group of the central tertiary nitrogen. Moreover, the molecular formula was determined to be C_44_H_49_N_3_O_5_ from the protonated molecular ion peak [M + H]^+^ found at *m*/*z* 700.3743 (700.3745 calcd for C_44_H_50_N_3_O_5_^+^). These prerequisites led to the identification of the structure shown in Figure 2. The framed substituents of the central tertiary nitrogen are identical; therefore, there is no reason for doubling the signals, as appeared in the 1D NMR data. Due to the steric hindrance of these two fragments, atropisomerism has been estimated as the reason for the doubling of some signals. The analysis was performed at ambient temperature and at 50 °C. The acquired results proved the suitability of this assumption. At ambient temperature, some signals were divided into two lines that broaden with temperature increase and eventually merge into a single line (see Supplementary Material). Unfortunately, the spectrum also revealed the signals of decomposition of this molecule.

### 2.2. Biological Activity Determination of Isolated Alkaloids

All the isolated compounds that had not been studied previously for their inhibition potential of cholinesterases, and which were obtained in sufficient amounts, were screened for their *h*AChE/*h*BuChE inhibition potency using a modified spectrophotometric method of Ellman et al. [36]. Furthermore, *h*AChE/*h*BuChE-active AAs were also studied for their ability to inhibit POP enzyme. Galanthamine and eserine were used as positive controls in the *h*AChE/*h*BuChE assays. Berberine was selected as a positive control when measuring POP inhibition. The results are summarized in Table 2. Moreover, the in vitro data are justified by docking studies proposing orientation of the top-ranked ligands within the *h*BuChE gorge.

The *h*AChE/*h*BuChE inhibitory activity of the AAs was initially screened at a concentration of 100 µM. Compounds displaying inhibition ability >50% against one or both cholinesterases at the screening concentration were selected for the determination of their IC_50_ values (Table 2).

**Figure 3:**
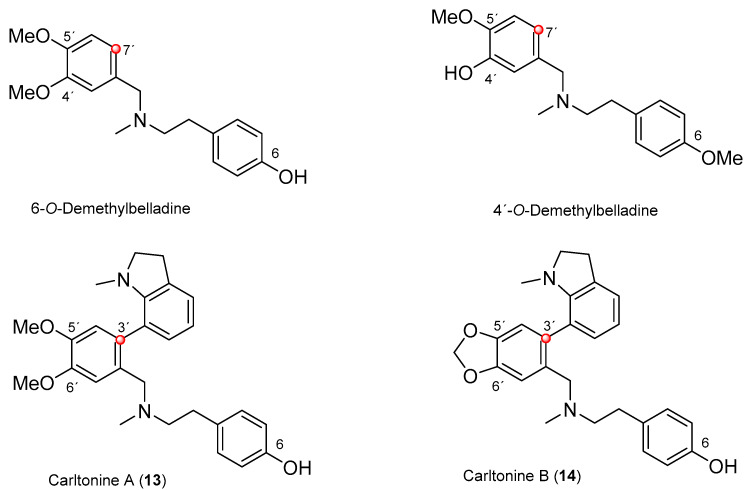
Structures of newly isolated (**13** and **14**) and recently reported belladine-type AAs 6-*O*-demethylbelladine and 4′-*O*-demethylbelladine [37].

In the *h*AChE assay, all studied AAs displayed marginal inhibition potency (Table 2). On the other hand, all newly isolated belladine-type alkaloids (**13**–**15**) demonstrated promising inhibition activity towards *h*BuChE (Table 2; Figure 3). Indeed, compounds **13** and **14** displayed IC_50_ values in the nanomolar range (*h*BuChE IC_50_ = 910 nM and 31 nM, for **13** and **14**, respectively).

From the structural perspective, both AAs are endowed with the same core structure, differing only in the substitution at positions C-5′ and C-6′, respectively (Figure 2). The presence of a 1,3-dioxolane ring in **14** compared with its opened dimethoxybenzene analogue **13** is plausibly responsible for the almost 30 times drop in *h*BuChE inhibition activity. Both compounds showed a *h*BuChE selective inhibition pattern with outstanding selectivity index (SI) values higher than 100 (Table 2).

Our group has previously isolated similar compounds from fresh bulbs of *Nerine bowdenii*: 6-*O*-demethylbelladine (**13**) and 4′-*O*-demethylbelladine (**14**) (Figure 3) [37]. However, neither of these two alkaloids are substituted at position C-7′, and differ from each other by the absence of one methoxy group (Figure 3) [37]. 4′-*O*-demethylbelladine (IC_50_ = 30.7 ± 4.0 µM) displayed slightly better in vitro inhibition activity of *h*BuChE compared with galanthamine (IC_50_ = 42 ± 1 µM). On the other hand, the compounds isolated within this study are more than 30 to 100 more potent *h*BuChE inhibitors, yielding a new structural lead scaffold that may be pursued in AD research.

Since some of the alkaloids were only isolated on a small scale, only two (**1** and **13**) were able to be tested for POP inhibition. Alkaloid **13** demonstrated POP inhibition in the same range as berberine (Table 2) [38].

The biological profiles of the other AAs (Figure 1) have already been determined within previous studies on Amaryllidaceae plants [21,39,40,41].

### 2.3. Docking Study of Carltonine A (***13***) and Carltonine B (***14***)

To reveal fundamental interactions for **13** and **14** within the *h*BuChE active site (PDB ID: 4BDS) [42], molecular docking studies were carried out, enabling better insight into the structural requirements for inhibition. Since both **13** and **14** are tertiary amines, which are protonated at physiological pH, they behave as *pseudo*-enantiomers. In this study, we attempted to predict more bioactive conformers of **13** and **14** based on their energy score and their topology within *h*BChE. To gain a more in-depth outlook into the overall ligand-enzyme complex, we are also displaying several comparative structure overlay between (*R*)-**13**-(*R*)-**14** (Figure S4A), (*S*)-**13**-(*R*)-**13** (Figure S4B), (*S*)-**13**-(*S*)-**14** (Figure S4C), and (S)-**14**-(*R*)-**14** (Figure S4D) *pseudo*-enantiomers (see Supplementary Material).

**Figure 4:**
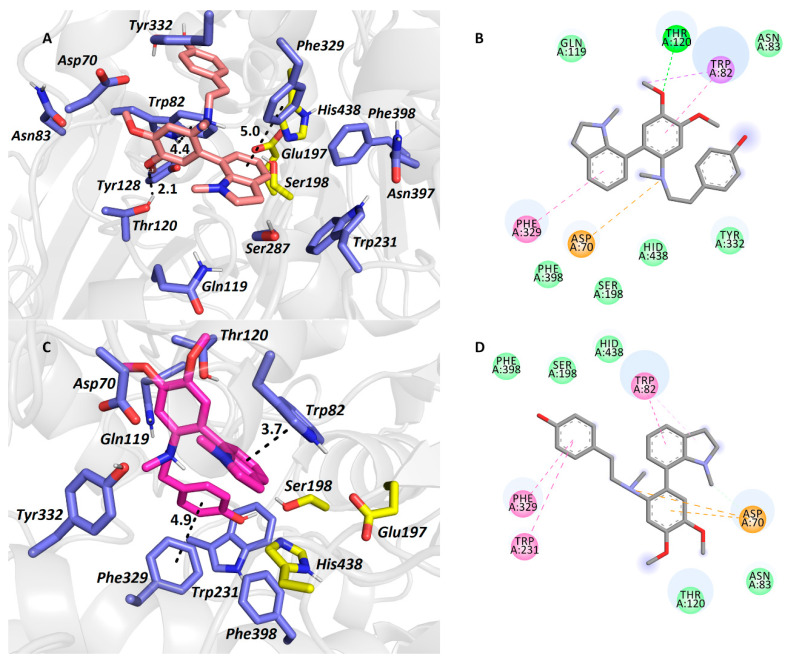
The *h*BuChE active site in complex with (*R*)-**13** (in salmon, **A**,**B**) and (*S*)-**13** (in purple, **C**,**D**) *pseudo*-enantiomers. All the amino acids exhibiting in the vicinity of the ligands up-to 6.0 Å are rendered. Hydrogen atoms of amino acids are omitted for clarity. Catalytic triad residues are shown in yellow, and amino acid residues in blue (**A**,**C**). In 2D diagrams (**B**,**D**), crucial amino acid residues are displayed in different colours depending on the nature of the interaction (e.g., purple for π-π, orange for anion-π, dark green for van der Waals contact, and light green for conventional hydrogen bond).

**Figure 5:**
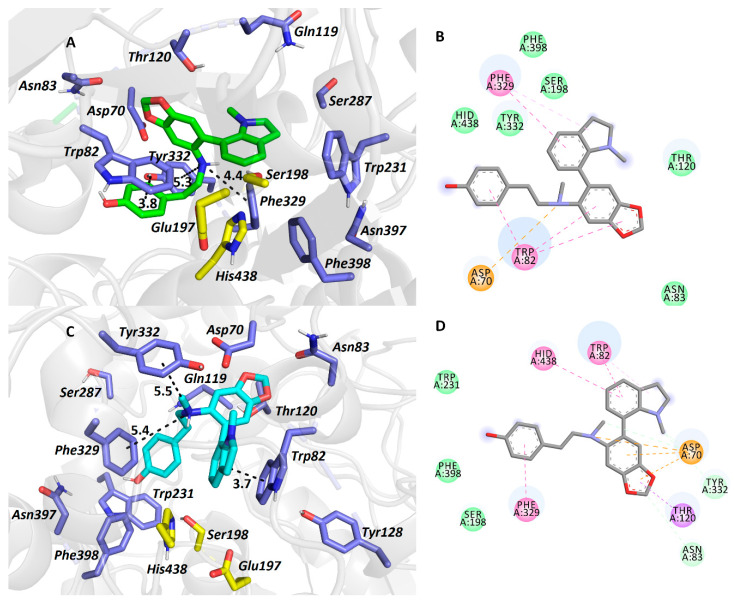
*h*BuChE active site in complex with (*R*)-**14** (in green, **A**,**B**) and (*S*)-**14** (in light-blue, **C**,**D**) *pseudo*-enantiomers. All the amino acids exhibiting in the vicinity of the ligands up-to 6.0 Å are rendered. Hydrogen atoms of amino acids are omitted for clarity. Catalytic triad residues are shown in yellow and amino acid residues in blue (**A**,**C**). In 2D diagrams (**B**,**D**), crucial amino acid residues are displayed in different colours depending on the nature of the interaction (e.g., purple for π-π, orange for anion-π, dark green for van der Waals contact, light green for conventional hydrogen bond).

Carltonine A (**13**) with a central amino moiety in (*R*) (Figure 4A,B) and (*S*) (Figure 4C,D) conformations displayed completely different binding modes, even though their estimated energetic balances were equipotent (−10.6 kcal/mol for both). For the (*R*)-**13** *pseudo*-enantiomer-*h*BuChE complex (Figure 5A,B), the *N*-methylindoline moiety occupies the vicinity of the catalytic triad with a T-shaped π-π interaction close to Phe329 (5.0 Å, distance measured from ring-to-ring center). Trp82 faces the dimethoxybenzene ring by displacing π-π interaction, whereas the phenolic appendage is left somewhat vacant. In the (*S*)-**13** *pseudo*-enantiomer-*h*BuChE complex (Figure 4C,D), the *N*-methylindoline moiety is engaged in parallel π-π stacking with Trp82 (3.7 Å). Contrary to the vacant occupancy of the phenolic appendage in the (*R*)-**13** *pseudo*-enantiomer, in the case of the (*S*)-**13** *pseudo*-enantiomer, Phe329 (4.9 Å) and Trp231 (5.5 Å) anchor this moiety. The dimethoxybenzene ring is stabilized by π-anion contact to Asp70.

The top-ranked docking poses for (*R*)-**14** and (*S*)-**14** *pseudo*-enantiomers in *h*BuChE active site are depicted in Figure 5A–D, respectively. In this case, binding energies differ favoring the (*R*)-**14** *pseudo*-enantiomer (−11.6 kcal/mol) over the (*S*)-**14** one (–10.9 kcal/mol). Similarly to the (*R*)-**13** *pseudo*-enantiomer-*h*BuChE complex, *N*-methylindoline is stabilized by T-shaped π-π interaction with Phe329 (5.0 Å) and by cation-π formation with the central nitrogen atom of (*R*)-**14** (Figure 5A,B). The protonated amino-group of (*R*)-**14** orchestrates the ligand-enzyme contact by other cation-π interactions with Tyr332 (5.3 Å) and Trp82 (4.5 Å). The latter amino acid residue is in close vicinity to 2*H*-1,3-benzodioxole displaying T-shaped π-π stacking (4.8 Å), and additionally to the phenolic moiety of the ligand via parallel π-π stacking (3.8 Å). Likewise, the same applies for the (*S*)-**14** *pseudo*-enantiomer in *h*BuChE (Figure 5C,D) when observing the central protonated amine of the ligand, i.e., cation-π contact with Tyr332 (5.5 Å) and Phe329 (5.4 Å), but not with Trp82. In this case, Trp82 revealed only an apparently parallel π-π interaction with the *N*-methylindoline moiety (3.7 Å).

Taken together from all the in silico observations above, it can be deduced that the higher inhibition potency of **14** over **13** might be attributed to the presence of the 2*H*-1,3-benzodioxole moiety for its “extended” aromatic properties that, especially in the (*R*)-**14** *pseudo*-enantiomer, broaden the range of hydrophobic interactions between the ligand and enzyme (Table 3).

## 3. Experimental

### 3.1. General Experimental Procedures

All solvents were treated using standard techniques before use. All reagents and catalysts were purchased from Sigma Aldrich, Czech Republic and used without purification. The NMR spectra were obtained in CDCl_3_ and CD_3_OD at ambient temperature on a VNMR S500 (Varian, Palo Alto, CA, USA) spectrometer operating at 500 MHz for ^1^H and 125.7 MHz for ^13^C. Chemical shifts were recorded as *δ* values in parts per million (ppm) and were indirectly referenced to tetramethylsilane (TMS) via the solvent signal (CDCl_3_—7.26 ppm for ^1^H and 77.0 ppm for ^13^C; CD_3_OD—3.30 ppm for ^1^H and 49.0 ppm for ^13^C). Coupling constants (*J*) are given in Hz. For unambiguous assignment of ^1^H and ^13^C signals, 2D NMR experiments, namely gCOSY, gHSQC, gHMBC and NOESY, were measured using standard parameter settings and standard pulse programs provided by the producer of the spectrometer. ESI-HRMS were obtained with a Waters Synapt G2-Si hybrid mass analyzer of a quadrupole-time-of-flight (Q-TOF) type, coupled to a Waters Acquity I-Class UHPLC system. The EI-MS were obtained on an Agilent 7890A GC 5975 inert MSD operating in EI mode at 70 eV (Agilent Technologies, Santa Clara, CA, USA). A DB-5 column (30 m × 0.25 mm × 0.25 μm, Agilent Technologies, USA) was used with a temperature program: 100–180 °C at 15 °C/min, 1 min hold at 180 °C, and 180–300 °C at 5 °C/min and 5 min hold at 300 °C; and detection range *m/z* 40–600. The injector temperature was 280 °C. The flow-rate of carrier gas (helium) was 0.8 mL/min. A split ratio of 1:15 was used. TLC was carried out on Merck precoated silica gel 60 F254 plates. Compounds on the plate were observed under UV light (254 and 366 nm) and visualized by spraying with Dragendorff’s reagent.

### 3.2. Plant Material

The fresh bulbs of *Narcissus pseudonarcissus* cv. Carlton were obtained from the herbal dealer Lukon Glads (Sadská, Czech Republic). Botanical identification was performed by Prof. L. Opletal. A voucher specimen is deposited in the Herbarium of the Faculty of Pharmacy in Hradec Králové under number: CUFPH-16130/AL-654.

### 3.3. Extraction and Isolation of Alkaloids

Fresh bulbs (30 kg) were minced and completely extracted with ethanol (EtOH) (96%, *v*/*v*, 3×) by boiling for 30 min under reflux; the combined extract was filtered and evaporated to dryness under reduced pressure. The crude extract (485 g) was acidified to pH 1–2 with 2% hydrochloric acid (HCl; 1 L) and the volume of the suspension was made up to 5 L with water. The suspension was filtered; the filtrate was defatted by diethyl ether (Et_2_O, 3 × 1.5 L), alkalized to pH 9–10 with a 10% solution of sodium carbonate (Na_2_CO_3_) and extracted with ethyl acetate (EtOAc; 3 × 1.5L). The organic layer was evaporated to give 245 g of dark brown fluid residue. The alkaloid summary extract was again dissolved in 2% HCl (1000 mL), washed with Et_2_O (3 × 300 mL) and alkalized to pH 9–10 with 10% Na_2_CO_3_. The water layer was extracted with Et_2_O (4 × 350 mL) and chloroform (CHCl_3_; 4 × 350 mL). Both Dragendorff positive parts were evaporated and pooled. A concentrated alkaloid extract (187 g) in the form of brown syrup was obtained.

The alkaloid extract was further fractionated by column chromatography on aluminum oxide (Al_2_O_3_; 5800 g), eluting with light petrol gradually enriched with CHCl_3_ (1:1, 2:3, 1:4; each 5000 mL), followed by CHCl_3_ (3000 mL) and finally by CHCl_3_ enriched with EtOH (99:1, 49:1, 97:3, 12:1, 1:1; 0:100 each 3000 mL). Fractions were collected in amounts of 1000 mL and monitored by TLC. Finally, 423 fractions were collected, combined into 25 fractions, and analyzed by GC-MS. Fractions with similar profile were pooled together to give nine final fractions (**I**–**IX**).

Fraction **I** (650 mg) was recrystallized from an ethanol/chloroform mixture (1:1, 100 mL) to give lycosinine B (**1**, 48 mg). Preparative TLC of the mother liquor (To: cHx: Et_2_NH, 60:40:5, 1×) led to the isolation of trispheridine (**2**, 5 mg). Repeated preparative TLC of the mother liquor gave 3,4-anhydrogalanthamine (**3**, 5 mg).

Fraction **II** (500 mg) was fractionated by preparative TLC (cHx: Me_2_CO: NH_3_; 30: 60: 1; 1×). Three subfractions were obtained (**IIa–c**). Repeated preparative TLC of subfraction **IIb** (cHx: Me_2_CO: NH_3_, 30: 60: 1; 2×) led to the isolation of oduline (**4**; 11 mg). Additional preparative TLC of subfraction **IIc** gave masonine (**5**; 44 mg).

Fraction **III** (16.3 g) was crystallized and recrystallized from EtOH and, finally, 6.39 g of galanthamine (**6**) was obtained.

Fraction **IV** (1.7 g) was further chromatographed by preparative TLC (To: cHx: Et_2_NH, 10:10:2, 2×) to obtain subfractions **Iva**–**b**. Repeated preparative TLC of **IVa** (320 mg; cHx: Et_2_NH, 9:1; 2×) led to the isolation of galanthine (**7**; 120 mg).

Fraction **V** (3.05 g) was crystallized and recrystallized three times from EtOH to give lycorenine (**9**; 981 mg).

Fraction **VI** (950 mg) was subjected to preparative TLC (To: cHx: Et_2_NH, 50:50: 5; 2×) to obtain four subfractions **VIa-d**. Subfraction **VIa** (120 mg) was treated by preparative TLC (cHx: Me_2_CO: NH_3_, 20:80:1; 2x) to give lycoramine (**9**; 20 mg). Subfraction **VIb** (150 mg) was subjected to preparative TLC (To: cHx: Et_2_NH; 40: 60: 5; 2×), crystallized from EtOH, and 45 mg of homolycorine (**10**) was obtained.

Fraction **VII** (25 g) was dissolved in hot EtOH and crystallized. Crude haemanthamine (**11;** 14.5 g) was obtained. This was repetitively (3×) recrystallized from hot EtOH to give 10.5 g of pure haemanthamine.

Repetitive preparative TLC (EtOAc: cHx: Et_2_NH; 70: 20: 10; 3×) of fraction **VIII** (3.25 g) led to the isolation of five subfractions (**VIIIa**-**e**). Subfraction **VIIIa** (545 mg) was subjected to preparative TLC (CH_3_CN: MeOH: NH_3;_ 70: 20: 0.3; 2×) to give vitattine (**12**; 150 mg). Subfraction **VIIIc** (550 mg) was chromatographed by preparative TLC (EtOAc: cHx: NH_3_; 40: 24: 0.6; 2×) to give carltonine A (**13**; 70 mg) and subfraction **VIIIc/1**. Subfraction VIIIc/1 (80 mg) was subjected to preparative TLC (EtOAc: cHx: NH_3_; 40: 24: 0.6; 1×) and carltonine B (**14**; 6 mg) was obtained. Subfraction **VIIId** (75 mg) was further treated by preparative TLC (EtOAc: Chx: NH_3_; 40: 24: 0.6; 2×) to yield carltonine C (**15**; 7 mg).

Fraction **IX** (1.65 g) was subjected to preparative TLC (To: Me_2_CO: MeOH: NH_3_; 50:60: 10:1; 1×) to give two subfractions **IXa**-**b**. Subfraction IXa (320 mg) was recrystallized from hot EtOH and separated by preparative TLC (To: Me_2_CO: NH_3_; 40:60:1; 2×) to give 9-*O-*demethylhomolycorine (**16**; 35 mg).

*Carltonine A (**13**)*: pale yellow oil; for ^1^H and ^13^C NMR data see Table 1; HRMS *m/z* 433.2488 [M + H]^+^ (calculated for C_27_H_33_N_2_O_3_^+^, 433.2486).

*Carltonine B (**14**)*: pale yellow oil; for ^1^H and ^13^C NMR data see Table 1; HRMS *m/z* 417.2172 [M + H]^+^ (calculated for C_26_H_29_N_2_O_3_^+^, 417.2173).

*Carltonine C (**15**)*: yellowish amorphous solid; for ^1^H and ^13^C NMR data see Table 1; HRMS *m/z* 700.3743 [M + H]^+^ (calculated for C_44_H_50_N_3_O_5_^+^, 700.3745).

### 3.4. Biological Assays

#### 3.4.1. hAChE and hBuChE Inhibition Assay

The *h*AChE and *h*BuChE activities were determined using a modified method of Ellman, as described [36,43,44]. Briefly, *h*AChE and *h*BuChE activities were determined using a modified method of Ellman, with acetylthiocholine iodide (ATChI) and butyrylthiocholine iodide (BuTChI) as substrates, respectively. Briefly, 8.3 μL of either blood cell lysate or plasma dilutions (at least six different concentrations), 283 μL of 5 mM 5,5′-dithiobis-2-nitrobenzoic acid (DTNB) and 8.3 μL of the sample dilution in dimethyl sulfoxide (DMSO) (40 mM, 10 mM, 4 mM, 1 mM, 0.4 mM, and 0 mM) were added to the semi-micro cuvette. The reaction was initiated by addition of 33.3 μL 10 mM substrate (ATChI or BuTChI). The final proportion of DTNB and substrate was 1:1. The increase of absorbance (ΔA) at 436 nm for AChE and 412 nm for BuChE was measured for 1 min at 37 °C using a spectrophotometer (Synergy^TM^ HT Multi-Detection Microplate Reader). Each measurement was repeated six times for every concentration of enzyme preparation. The % inhibition was calculated according to the following formula:

(1) $$\%\;I=100-\left(100\times \frac{\Delta A_{Bl}}{\Delta A_{Sa}}\right),$$

where Δ*A_Bl_* is the increase of absorbance of the blank sample and Δ*A_Sa_* is the increase of absorbance of the measured sample. Inhibition potency of the tested compounds was expressed as an IC_50_ value (concentration of inhibitor, which causes 50% cholinesterase inhibition).

#### 3.4.2. POP Inhibition Assay

POP (EC 3.4.21.26) was dissolved in phosphate-buffered saline (PBS; 0.01 M Na/K phosphate buffer, pH 7.4, containing 137 mM NaCl and 2.7 mM KCl); the specific activity of the enzyme was 0.2 U/mL. The assay was performed in standard polystyrene 96-well microplates with a flat and clear bottom. Stock solutions of tested compounds were prepared in DMSO (10 mM). Dilutions (10^−3^ to 10^−7^ M) were prepared from the stock solution with deionized H_2_O; the control was performed with the same DMSO concentration. POP substrate, (*Z*)-Gly-Pro-p-nitroanilide, was dissolved in 50% 1,4-dioxane (5 mM). For each reaction, PBS (170 μL), tested compound (5 μL), and POP (5 μL) were incubated for 5 min at 37 °C. Then, substrate (20 μL) was added, and the microplate was incubated for 30 min at 37 °C. The formation of p-nitroanilide, directly proportional to the POP activity, was measured spectrophotometrically at 405 nm using a microplate ELISA reader (multimode microplate reader Synergy 2, BioTek Instruments, Winooski, VT, USA). The inhibition potency of tested compounds was calculated by nonlinear regression analysis and was expressed as an IC_50_ value (concentration of inhibitor which causes 50% POP inhibition). All calculations were performed using GraphPad Prism software version 7.03 for Windows (GraphPad Software Inc., San Diego, California, USA) [44].

#### 3.4.3. Molecular Modelling Studies

Two structures of *h*AChE and *h*BuChE were gained from RCSB Protein Data Bank—PDB ID: 4EY6 (crystal structure of *h*AChE) and 4BDS (crystal structure of *h*BuChE) [42,45]. All receptor structures were prepared by DockPrep function of UCSF Chimera (v. 1.4) and converted to pdbqt-files by AutodockTools (v. 1.5.6) [46,47]. Flexible residues selection was based on previous experience with either *h*AChE, *h*BuChE or the spherical region around the binding cavity [48,49,50]. Three-dimensional structures of ligands were built by Open Babel (v. 2.3.1), minimized by Avogadro (v 1.1.0) and converted to pdbqt-file format by AutodockTools [51]. The docking calculations were made by Autodock Vina (v. 1.1.2) with the exhaustiveness of 8 [52]. Calculation was repeated 20 times for each ligand and receptor and the best-scored result was selected for manual inspection. The visualization of enzyme-ligand interactions was prepared using The PyMOL Molecular Graphics System (Version 2.0, Schrödinger, LLC, Mannheim, Germany). 2D diagrams were created with Dassault Systèmes BIOVIA, Discovery Studio Visualizer (v 17.2.0.16349, Dassault Systèmes, 2016, San Diego, CA, USA).

## 4. Conclusions

The phytochemical study of the alkaloidal extract of *Narcissus pseudonarcissus* cv. Carlton resulted in the isolation of thirteen previously described AAs, and three new AAs of belladine-type, named carltonine A–C (**13**-**15**). Their structures were elucidated using a combination of NMR and MS analysis. Compounds isolated in sufficient quantity and not previously tested for their biological activities in relation to AD, were screened for their potential to inhibit *h*AChE, *h*BuChE and POP. Significant and selective *h*BuChE inhibitory activity was demonstrated by the newly described alkaloids carltonine A (**13**) and carltonine B (**14**) with IC_50_ values of 0.91 ± 0.02 µM and 0.031 ± 0.001 µM, respectively. The in vitro results were justified by computational studies predicting plausible binding modes of compounds **13** and **14** in the active site of *h*BuChE. The new compounds exerted an interesting biological profile deserving further lead-optimization. The next step will be the development of an appropriate synthetic route leading to carltonine derivatives with follow-up preparation of semi-synthetic derivatives.

## Figures and Tables

**Table 1:** ^1^H NMR (500 MHz) and ^13^C NMR (125.7 MHz) data for **13**–**15** in CDCl_3_ (*δ* in ppm and *J* in Hz).

	Carltonine A (13)		Carltonine B (14)		Carltonine C (15)	
Position	*δ*_C_	*δ*_H_	*δ*_C_	*δ*_H_	*δ*_C_	*δ*_H_
1	59.2	2.58–2.46, m	59.3	2.56–2.45, m	55.2; 55.4	2.66–2.42, m
2	32.8	2.65, t (7.4)	32.7	2.66–2.58, m	32.7; 32.8	2.66–2.42, m
3	132.5		132.2		132.8	
4	129.8	7.00–6.96, m	129.7	7.00–6.94, m	129.9	6.93–6.86, m
5	115.1	6.72–6.68, m	115.2	6.75–6.72, m	114.9	6.71–6.65, m
6	154.0		153.9		153.7	
7	115.1	6.72–6.68, m	115.2	6.75–6.72, m	114.9	6.71–6.65, m
8	129.8	7.00–6.96, m	129.7	7.00–6.94, m	129.9	6.93–6.86, m
1′	58.5	3.45, d (14.0) 3.29, d, overlap (14.0)	58.3	3.46, d (13.2) 3.27, d (13.2)	55.1; 55.0	3.43, d (14.5) 3.29, d (14.5); 3.37, s
2′	130.1		130.6		131.2; 131.0	
3′	132.1		133.3		131.9; 131.8	
4′	113.2	6.76, s	110.2	6.75–6.72, m	113.23; 113.15	6.74, s
5′	146.9		145.9		146.7	
6′	148.1		147.0		147.9	
7′	111.1	7.10, s, overlap	108.6	7.07, s, overlap	110.6; 110.5	7.15, s; 7.14, s
2″	57.1	3.32, dt, overlap (17.3, 8.8) 3.20, dt (17.3, 8.8)	57.1	3.35, dt (17.5, 8.7) 3.16, dt (17.5, 8.7)	57.1; 57.0	3.27–3.17, m
3″	28.6	2.98, t (8.8)	28.6	3.00–2.93, m	28.6	2.99–2.91, m
3a″	131.2		131.2		131.1	
4″	123.3	7.09, dd, overlap (7.4, 1.0)	123.4	7.08, d, overlap (7.4)	123.3	7.07, d (7.4)
5″	117.9	6.72, t, overlap (7.4)	118.1	6.70, t, overlap (7.4)	117.9; 117.8	6.71–6.65, m
6″	130.5	6.82, dd (7.4, 1.0)	130.4	6.79, d (7.4)	130.3; 130.4	6.77, d (7.4)
7″	123.0		122.9		122.8	
7a″	150.4		150.4		150.3; 150.2	
N-Me	42.3	2.20, s	41.9	2.18, s	-	-
N1″-Me	38.6	2.21, s	38.8	2.23, s	38.51; 38.48	2.14, s; 2.12, s
5′-OMe	55.8 or 55.9	3.34, s	-	-	55.9	3.84, s
6′-OMe	55.8 or 55.9	3.34, s	-	-	55.6	3.82, s; 3.81, s
-OCH_2_O-	-	-	101.0	5.99, s 5.98, s	-	-

**Table 2:** In vitro results of *h*AChE, *h*BuChE and POP inhibition of selected AAs isolated from *Narcissus pseudonarcissus* cv. Carlton.

Compound	%Inhibition *h*AChE ± SD ^a^	*h*AChE IC_50_ ± SD (µM) ^b^	% inhibition *h*BuChE ± SD ^a^	*h*BuChE IC_50_ ± SD (µM) ^b^	SI for *h*BuChE ^c^	POP IC_50_ ± SD (µM) ^b^
Lycosinine B (**1**)	28 ± 1	>100	42 ± 1	>100	nc	258 ± 14
Trispheridine (**2**)	6 ± 1	>100	13 ± 1	>100	nc	nm
3,4-Anhydrogalanthamine (**3**)	4 ± 0	>100	28 ± 1	>100	nc	nm
Carltonine A (**13**)	2 ± 0	>100	98 ± 1	0.91 ± 0.02	>110	143 ± 12
Carltonine B (**14**)	40 ± 1	>100	99 ± 1	0.031 ± 0.001	>3226	nm
Carltonine C (**15**)	9 ± 0	>100	78 ± 1	14.8 ± 1.1	>7	nm
Galanthamine ^d^	nm	1.72 ± 0.12	nm	42 ± 1	0.04	nm
Eserine ^d^	nm	0.063 ± 0.005	nm	0.13 ± 0.01	0.48	nm
Berberine ^d^	nm	0.72 ± 0.11	nm	31 ± 4	0.02	142 ± 21

^a^ Tested at 100 µM compound concentration; ^b^ Compound concentration required to decrease enzyme activity by 50%; the values are the mean values ± standard deviations (SD) of three independent measurements, each performed in triplicate; ^c^ Selectivity index (SI) for *h*BuChE is determined as ratio *h*AChE IC_50_/*h*BuChE IC_50_; ^d^ Reference compound; nc stands for not calculated; nm stands for not measured.

**Table 3:** The best obtained calculated binding energies with Autodock Vina software for the carltonine derivatives under the in silico study within *h*BuChE enzyme.

Carltonine Enantiomer	Calculated Binding Energy (kcal/mol)
(*R*)-**13**	−10.6
(*S*)-**13**	−10.6
(*R*)-**14**	−11.6
(*S*)-**14**	−10.9

## Supplementary Materials

The following are available online at https://www.mdpi.com/2218-273X/10/5/800/s1, Figure S1-1: ESI-HRMS spectrum of carltonine A (**13**); Figure S1-2: ^1^H NMR spectrum of carltonine A (**13**) in CDCl_3_; Figure S1-3: ^13^C NMR spectrum of carltonine A (**13**) in CDCl_3_; Figure S1-4: gCOSY spectrum of carltonine A (**13**) in CDCl_3_; Figure S1-5: gHSQC spectrum of carltonine A (**13**) in CDCl_3_; Figure S1-6: gHMBCAD spectrum of carltonine A (13) in CDCl_3_; Figure S1-7: gH2BC spectrum of carltonine A (**13**) in CDCl_3_; Figure S2-1: ESI-HRMS spectrum of carltonine B (**14**); Figure S2-2: ^1^H NMR spectrum of carltonine B (**14**) in CDCl_3_; Figure S2-3: ^13^C NMR spectrum of carltonine B (**14**) in CDCl_3_; Figure S2-4: gCOSY spectrum of carltonine B (**14**) in CDCl_3_; Figure S2-5: gHSQC spectrum of carltonine B (**14**) in CDCl_3_; Figure S2-6: gHMBCAD spectrum of carltonine B (**14**) in CDCl_3_; Figure S2-7: gH2BC spectrum of carltonine B (**14**) in CDCl_3_; Figure S3-1: ESI-HRMS spectrum of carltonine C (**15**); Figure S3-2: ^1^H NMR spectrum of carltonine C (**15**) in CDCl_3_; Figure S3-3: ^13^C NMR spectrum of carltonine C (**15**) in CDCl_3_; Figure S3-4: gCOSY spectrum of carltonine C (**15**) in CDCl_3_; Figure S3-5: gHSQC spectrum of carltonine C (**15**) in CDCl_3_; Figure S3-6: gHMBCAD spectrum of carltonine C (**15**) in CDCl_3_; Figure S3-7: gH2BC spectrum of carltonine C (**15**) in CDCl_3_; Figure S3-8: ^1^H NMR spectrum of carltonine C (**15**) in CDCl_3_ at 50 °C; Figure S3-9: ^13^C NMR spectrum of carltonine C (**15**) in CDCl_3_ at 50 °C; Figure S4**:** Overlapped *pseudo*-enantiomers in the *h*BuChE active site and their topology difference.
