# Supplementary material for: Amaryllidaceae Alkaloids of Belladine-Type from Narcissus pseudonarcissus cv. Carlton as New Selective Inhibitors of Butyrylcholinesterase
Source: Biomolecules. 2020 May 22;10(5):800. doi: 10.3390/biom10050800 (PMC7277649; doi:10.3390/biom10050800)
Supplement: Supplementary file 1 [file biomolecules-10-00800-s001.pdf]

Supplementary Material

Amaryllidaceae alkaloids of belladine-type from *Narcissus pseudonarcissus* cv. Carlton as new selective inhibitors of butyrylcholinesterase

Abdullah Al Mammun[1], Jana Maříková[2], Daniela Hulcová[1,3], Jiří Janoušek[1,3], Marcela Šafratová[1,3], Lucie Nováková[4], Tomáš Kučera[5], Martina Hrabinová[6,7], Jiří Kuneš[2], Jan Korábečný[6,7,*], Lucie Cahlíková[1,*]

[1] ADINACO Research Group, Department of Pharmaceutical Botany, Faculty of Pharmacy, Charles University, Heyrovského 1203, 500 05 Hradec Králové, Czech Republic; almamuna@faf.cuni.cz (AAM)
[2] Department of Organic and Bioorganic Chemistry, Faculty of Pharmacy, Charles University, Heyrovského 1203, 500 05 Hradec Králové, Czech Republic; marikoj2@faf.cuni.cz (JM); kunes@faf.cuni.cz (JK)
[3] Department of Pharmacognosy, Faculty of Pharmacy, Charles University, Heyrovského 1203, 500 05 Hradec Králové, Czech Republic; hulcovd@faf.cuni.cz (DH); safratom@faf.cuni.cz (MS); janousj2@faf.cuni.cz (JJ)
[4] Department of Analytical Chemistry, Faculty of Pharmacy, Charles University, Heyrovského 1203, 500 05 Hradec Králové, Czech Republic; novakoval@faf.cuni.cz (LN)
[5] Department of Military Medical Service Organisation and Management, Faculty of Military Health Sciences, University of Defence, Třebešská 1575, 500 05 Hradec Králové, Czech Republic; kucera-t@email.cz (TK)
[6] Department of Toxicoloxy and Military Pharmacy, Faculty of Military Health Sciences, University of Defence, Třebešská 1575, 500 05 Hradec Králové, Czech Republic; hrabinova@pmfhk.cz (MH)
[7] Biomedical Research Centre, University Hospital Hradec Kralove, Sokolska 581, 500 05 Hradec Kralove, Czech Republic

* Correspondence: cahlikova@faf.cuni.cz; Tel.: + 420 495 067 311; ORCID: 0000-0002-1555-8870 (LC); jan.korabecny@fnhk.cz; Tel.: + 420 495 833 447; ORCID: 0000-0001-6977-7596 (JKO)

<u>Table of contents</u>

Figure S1-1. ESI-HRMS spectrum of carltonine A (**13**)........ 3

Figure S1-2. ^{1}H NMR spectrum of carltonine A (**13**) in........ 3

Figure S1-3. ^{13}C NMR spectrum of carltonine A (**13**) in $CDCl_3$........ 4

Figure S1-4. gCOSY spectrum of carltonine A (**13**) in $CDCl_3$........ 5

Figure S1-5. gHSQC spectrum of carltonine A (**13**) in $CDCl_3$........ 6

Figure S1-6. gHMBCAD spectrum of carltonine A (13) in........ 7

Figure S1-7. gH2BC spectrum of carltonine A (**13**) in........ 7

Figure S2-1. ESI-HRMS spectrum of carltonine B (**14**)........ 8

Figure S2-2. ^{1}H NMR spectrum of carltonine B (**14**) in........ 8

Figure S2-3. ^{13}C NMR spectrum of carltonine B (**14**) in $CDCl_3$........ 8

Figure S2-4. gCOSY spectrum of carltonine B (**14**) in $CDCl_3$........ 9

Figure S2-5. gHSQC spectrum of carltonine B (**14**) in $CDCl_3$........ 9

Figure S2-6. gHMBCAD spectrum of carltonine B (**14**) in........ 10

Figure S2-7. gH2BC spectrum of carltonine B (**14**) in........ 10

Figure S3-1. ESI-HRMS spectrum of carltonine C (**15**)........ 11

Figure S3-2. ^{1}H NMR spectrum of carltonine C (**15**) in $CDCl_3$........ 11

Figure S3-3. ^{13}C NMR spectrum of carltonine C (**15**) in $CDCl_3$........ 12

Figure S3-4. gCOSY spectrum of carltonine C (**15**) in $CDCl_3$........ 12

Figure S3-5. gHSQC spectrum of carltonine C (**15**) in $CDCl_3$........ 13

Figure S3-6. gHMBCAD spectrum of carltonine C (**15**) in $CDCl_3$........ 13

Figure S3-7. gH2BC spectrum of carltonine C (**15**) in $CDCl_3$........ 14

Figure S3-8. ^{1}H NMR spectrum of carltonine C (**15**) in $CDCl_3$ at 50 °C........ 14

Figure S3-9. ^{13}C NMR spectrum of carltonine C (**15**) in $CDCl_3$ at 50 °C........ 15

Figure S4 Overlapped *pseudo*-enantiomers in the *h*BuChE active site and their topology difference: (*R*)-**13**-(*R*)-**14** (A), (*S*)-**13**-(*R*)-**13** (B), (*S*)-**13**-(*S*)-**14** (C), and (S)-**14**-(*R*)-**14** (D). (*R*)-**13**, (*S*)-**13**, (*R*)-**14**, and (*S*)-**14** are shown in salmon, purple, green, and light blue, respectively. Catalytic triad residues are portrayed in yellow........ 16

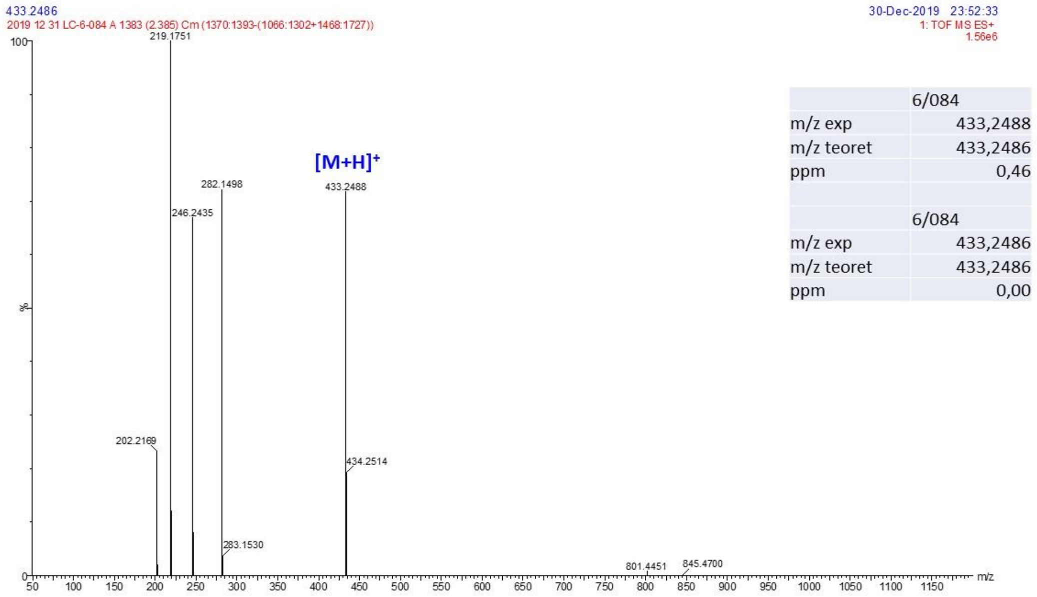

Figure S1-1. ESI-HRMS spectrum of carltonine A (**13**).

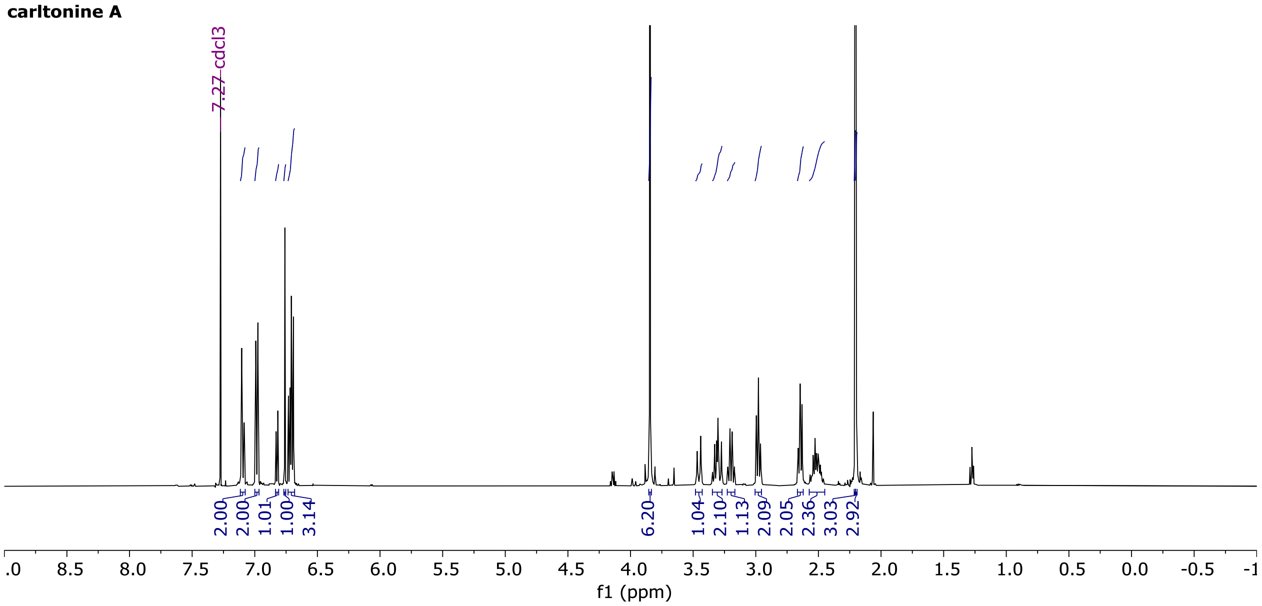

Figure S1-2. ¹H NMR spectrum of carltonine A (**13**) in CDCl3.

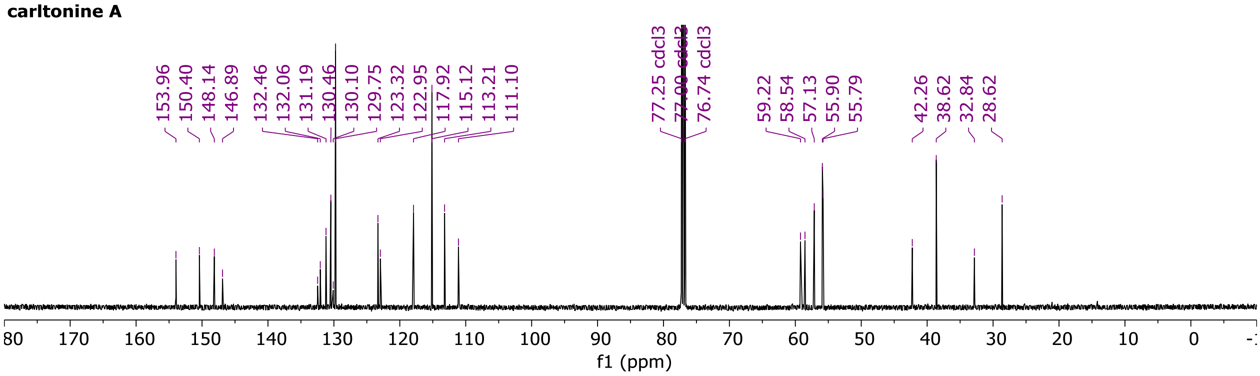

Figure S1-3. ^{13}C NMR spectrum of carltonine A (**13**) in $CDCl_3$.

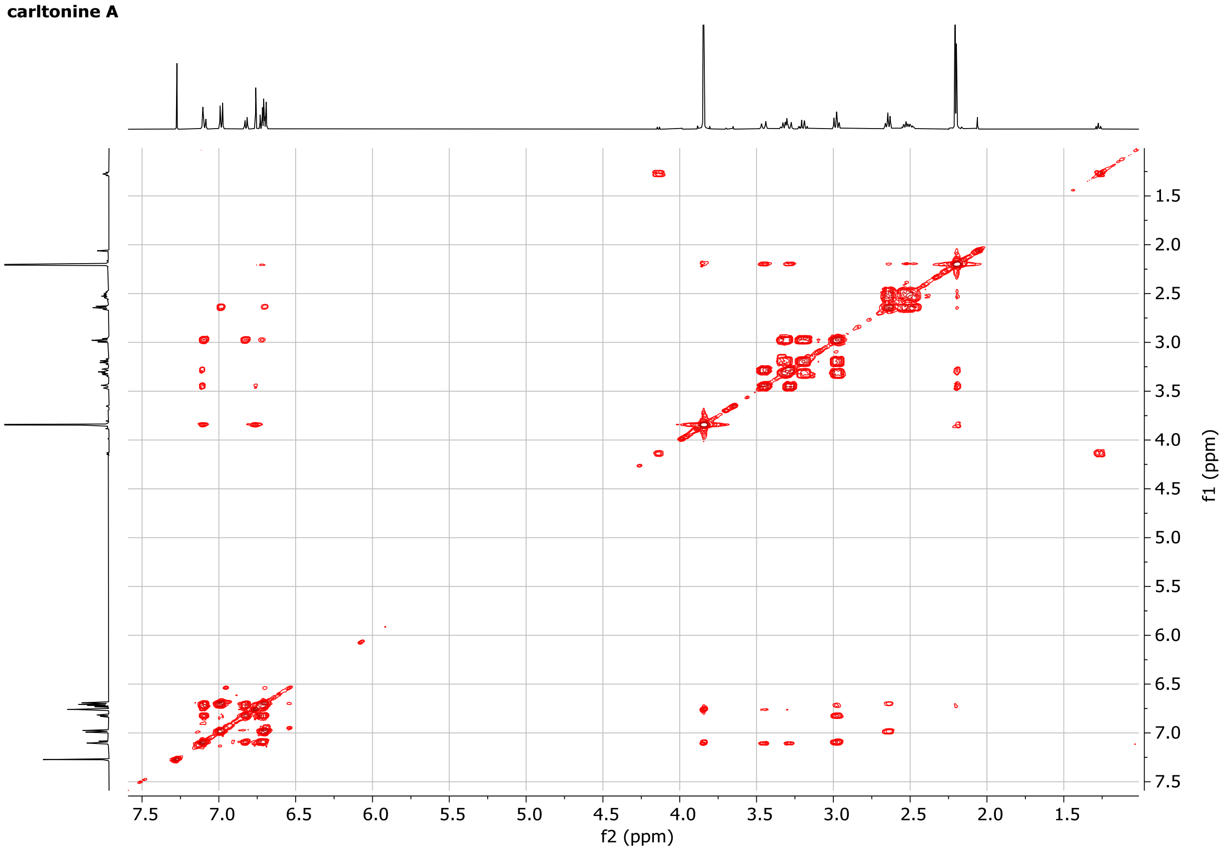

Figure S1-4. gCOSY spectrum of carltonine A (**13**) in $CDCl_3$.

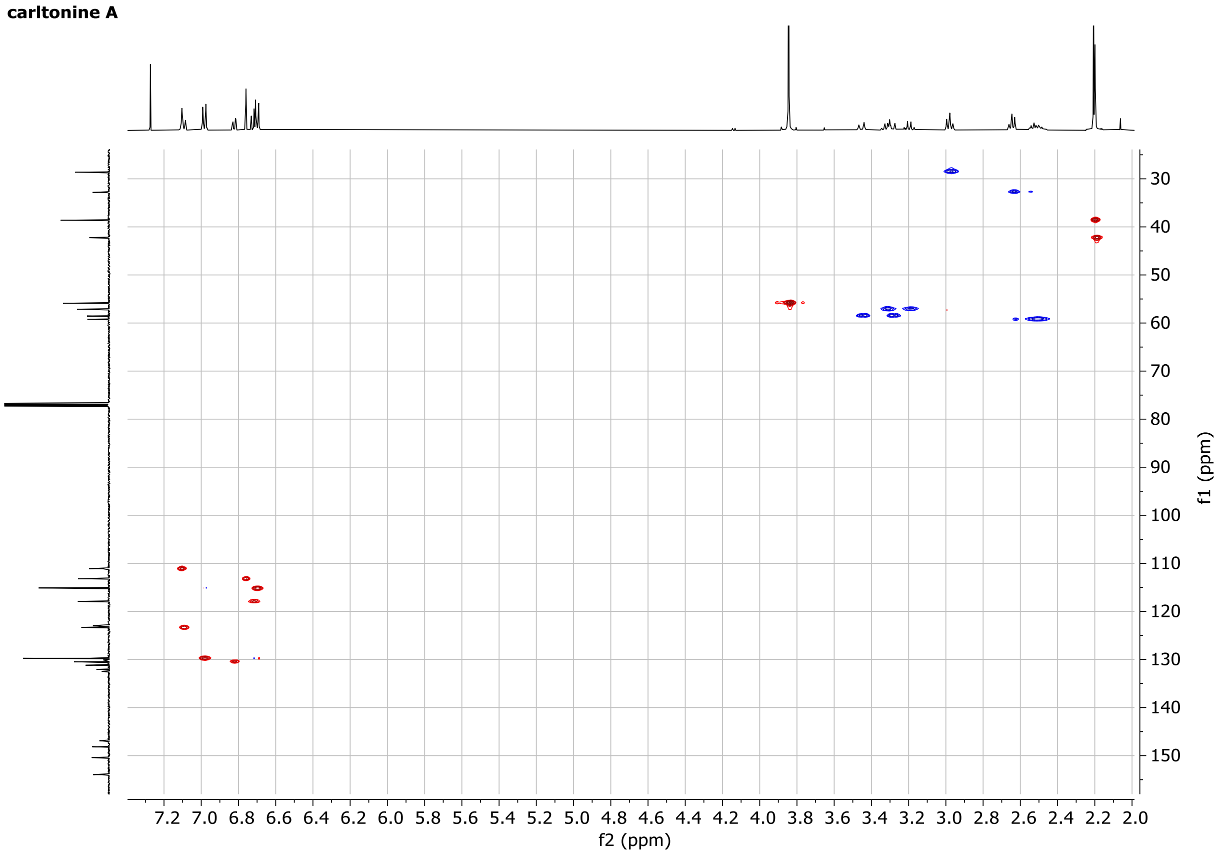

Figure S1-5. gHSQC spectrum of carltonine A (**13**) in $CDCl_3$.

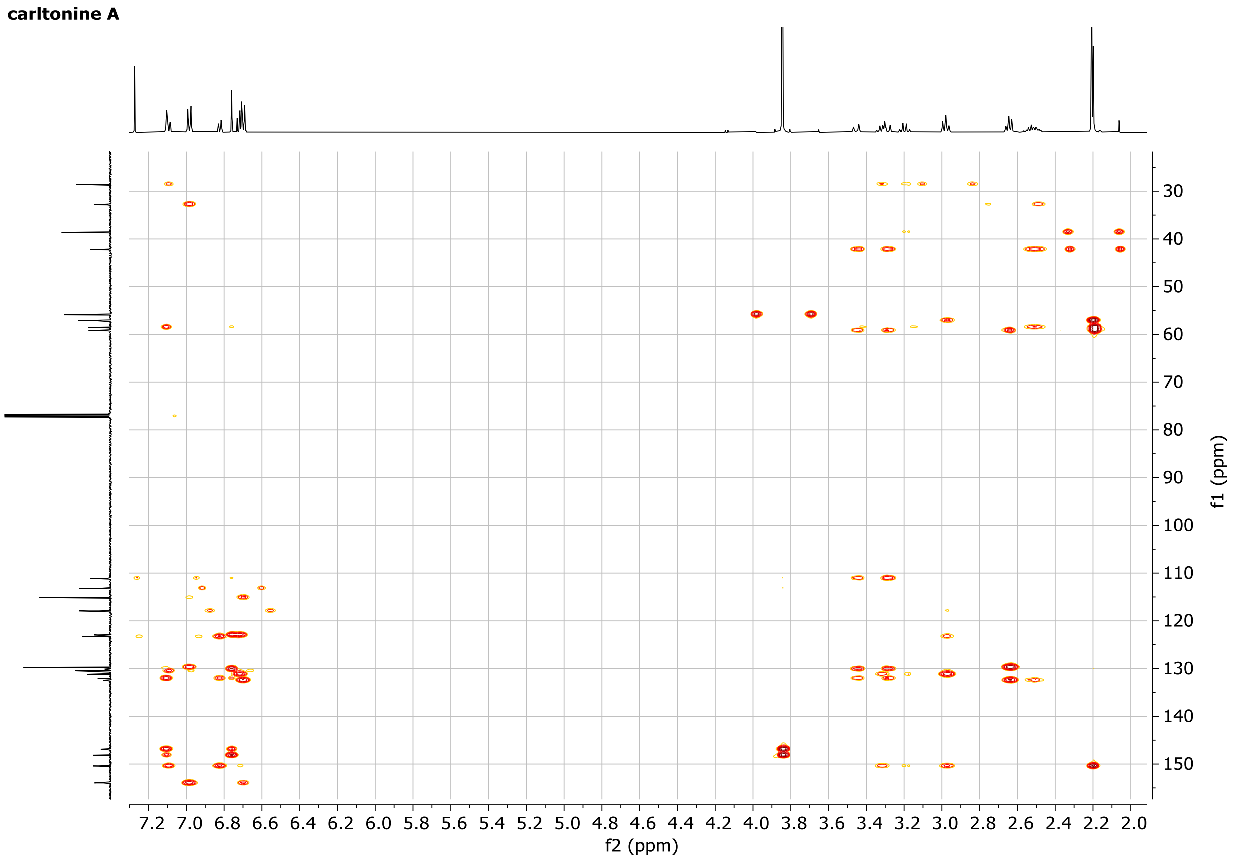

Figure S1-6. gHMBCAD spectrum of carltonine A (13) in $CDCl_3$.

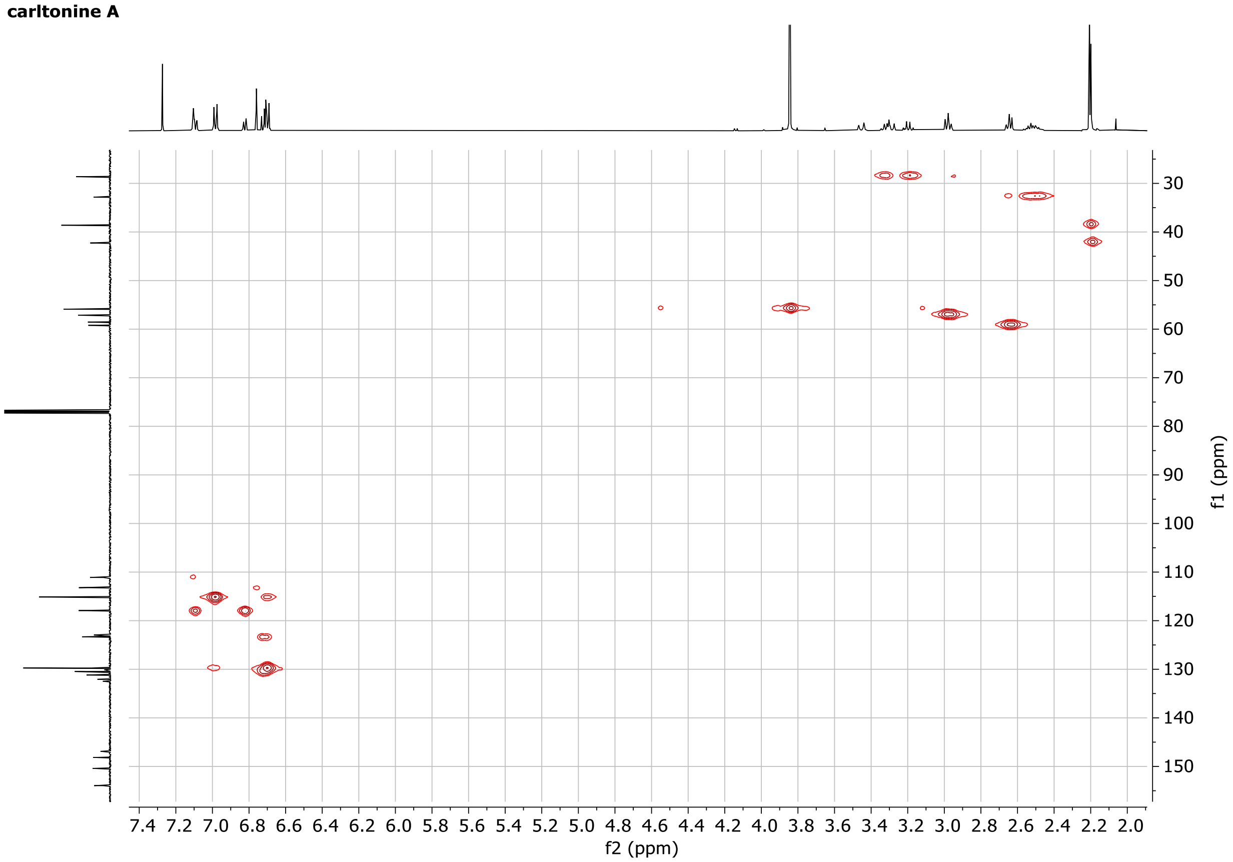

Figure S1-7. gH2BC spectrum of carltonine A (**13**) in $CDCl_3$.

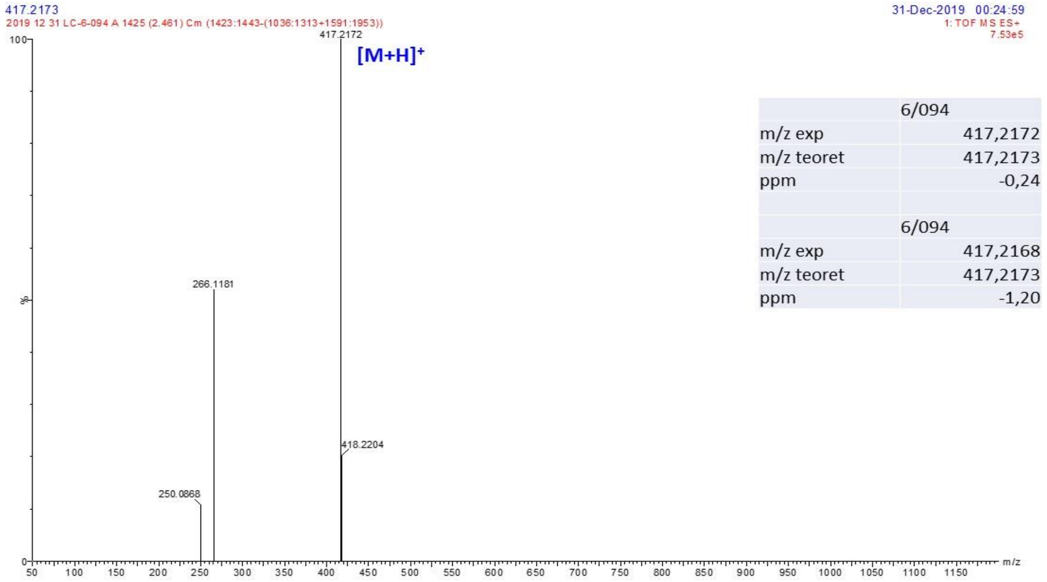

Figure S2-1. ESI-HRMS spectrum of carltonine B (**14**).

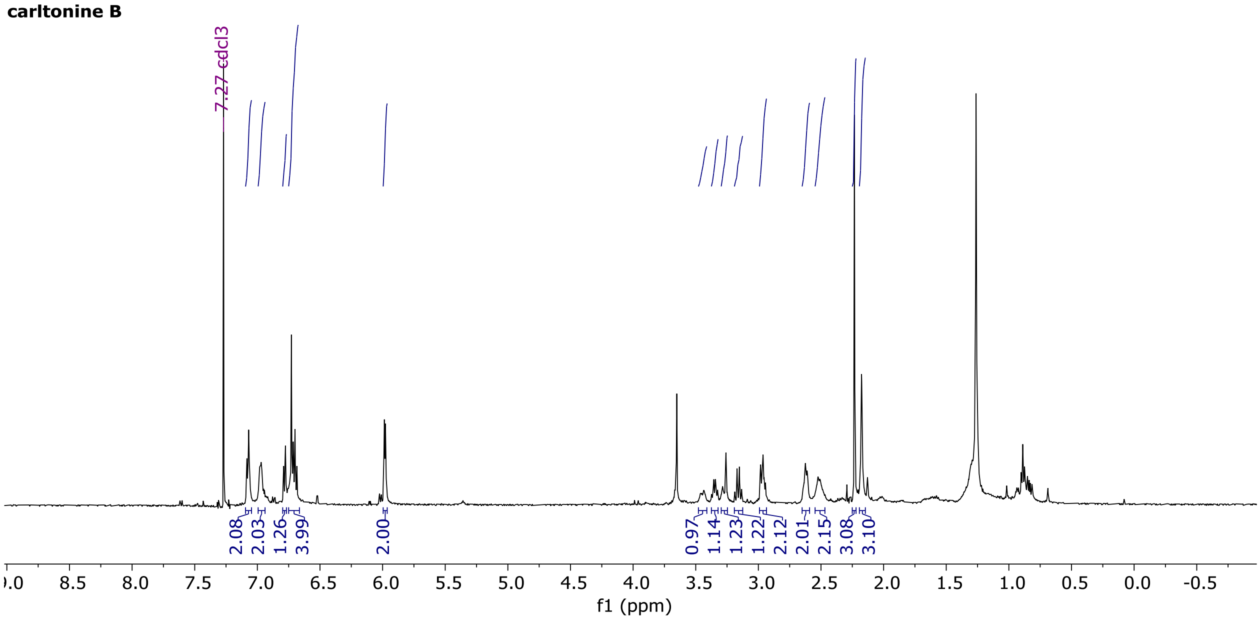

Figure S2-2. ^{1}H NMR spectrum of carltonine B (**14**) in $CDCl_3$.

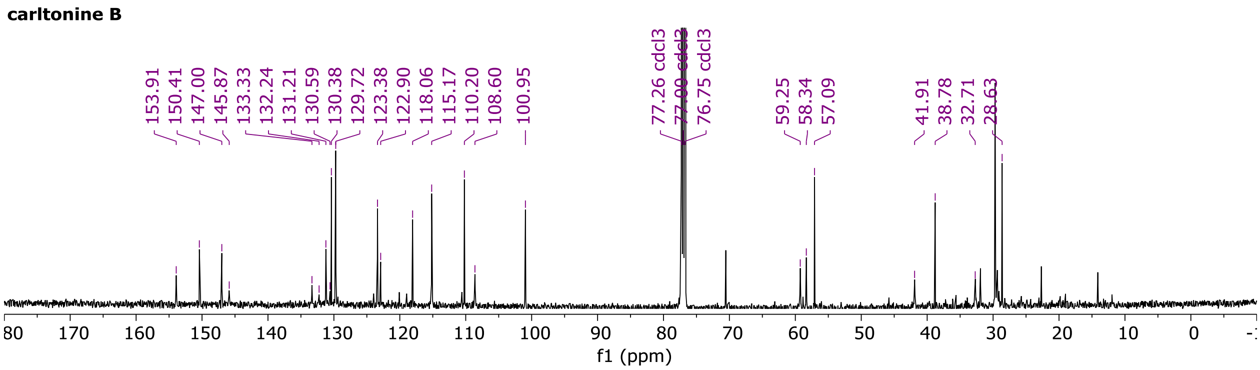

Figure S2-3. ^{13}C NMR spectrum of carltonine B (**14**) in $CDCl_3$.

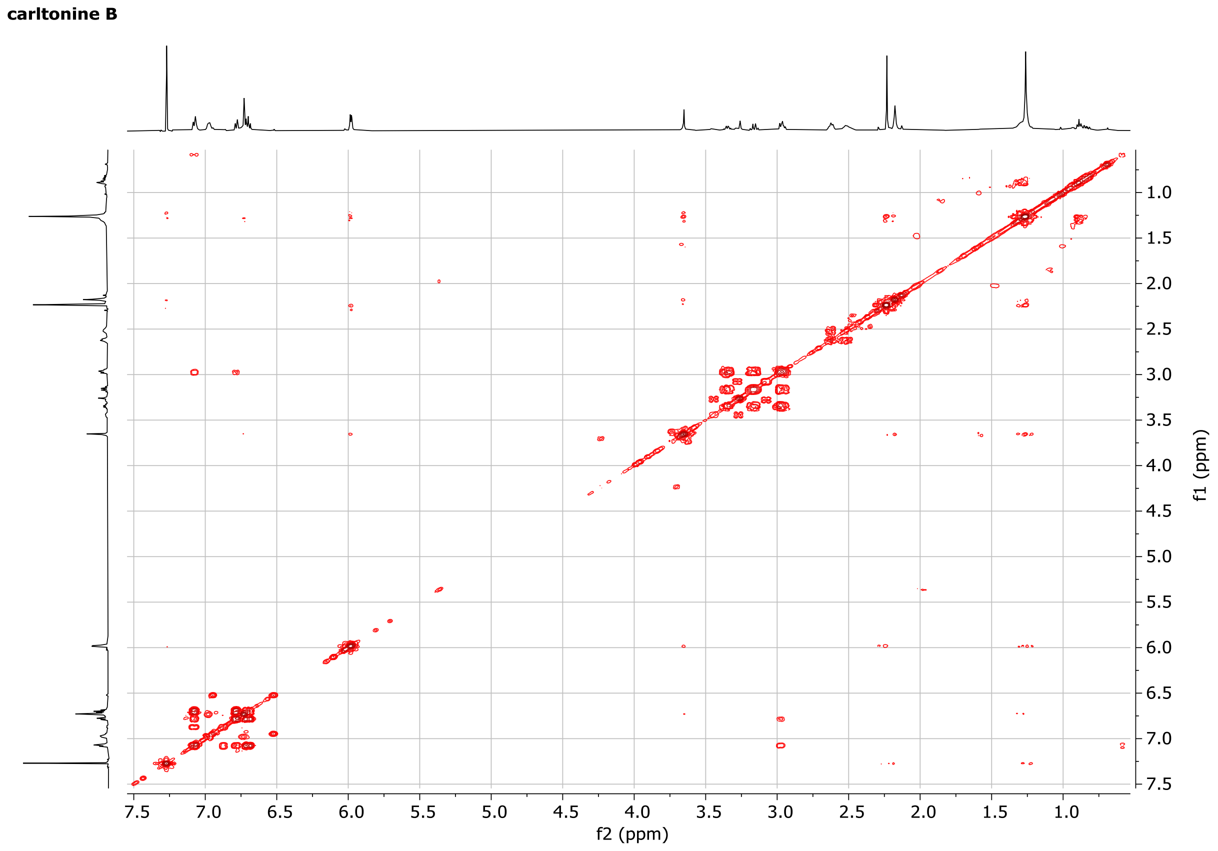

Figure S2-4. gCOSY spectrum of carltonine B (**14**) in $CDCl_3$.

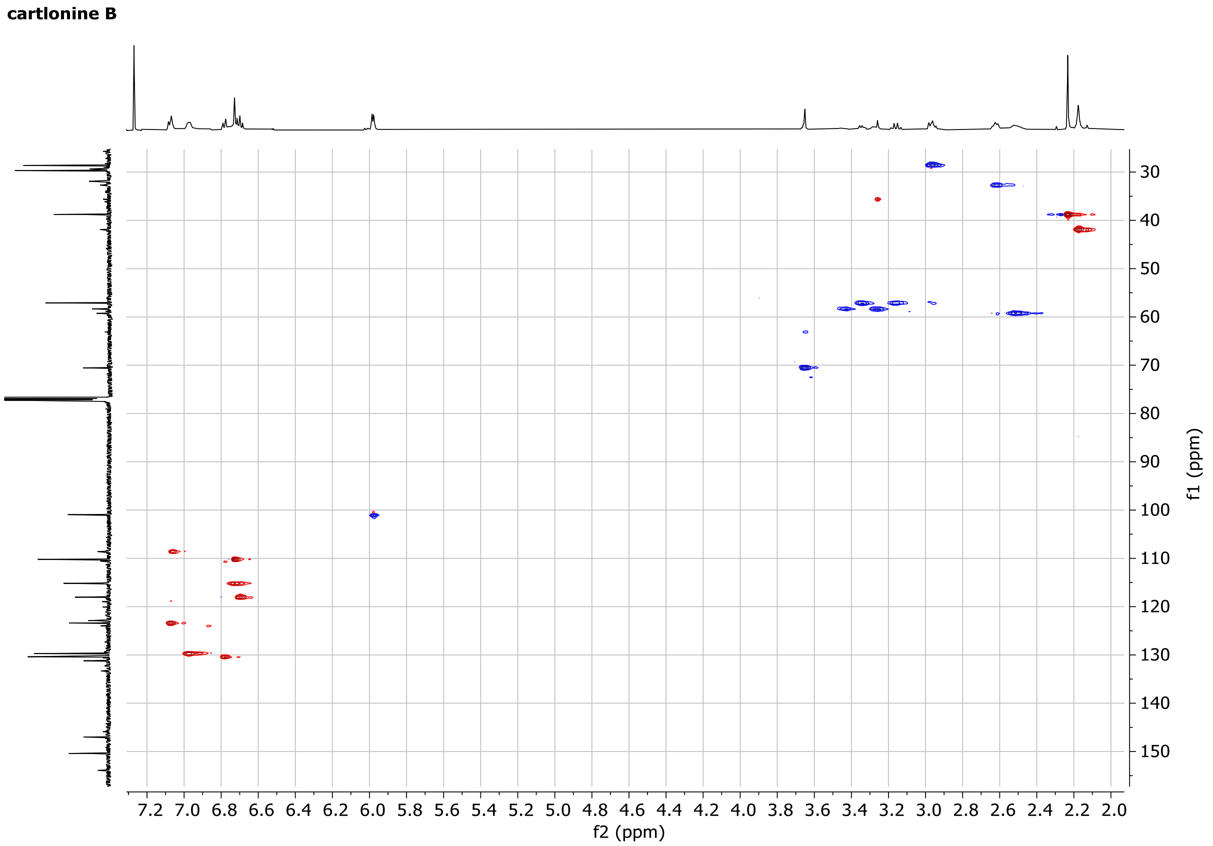

Figure S2-5. gHSQC spectrum of carltonine B (**14**) in $CDCl_3$.

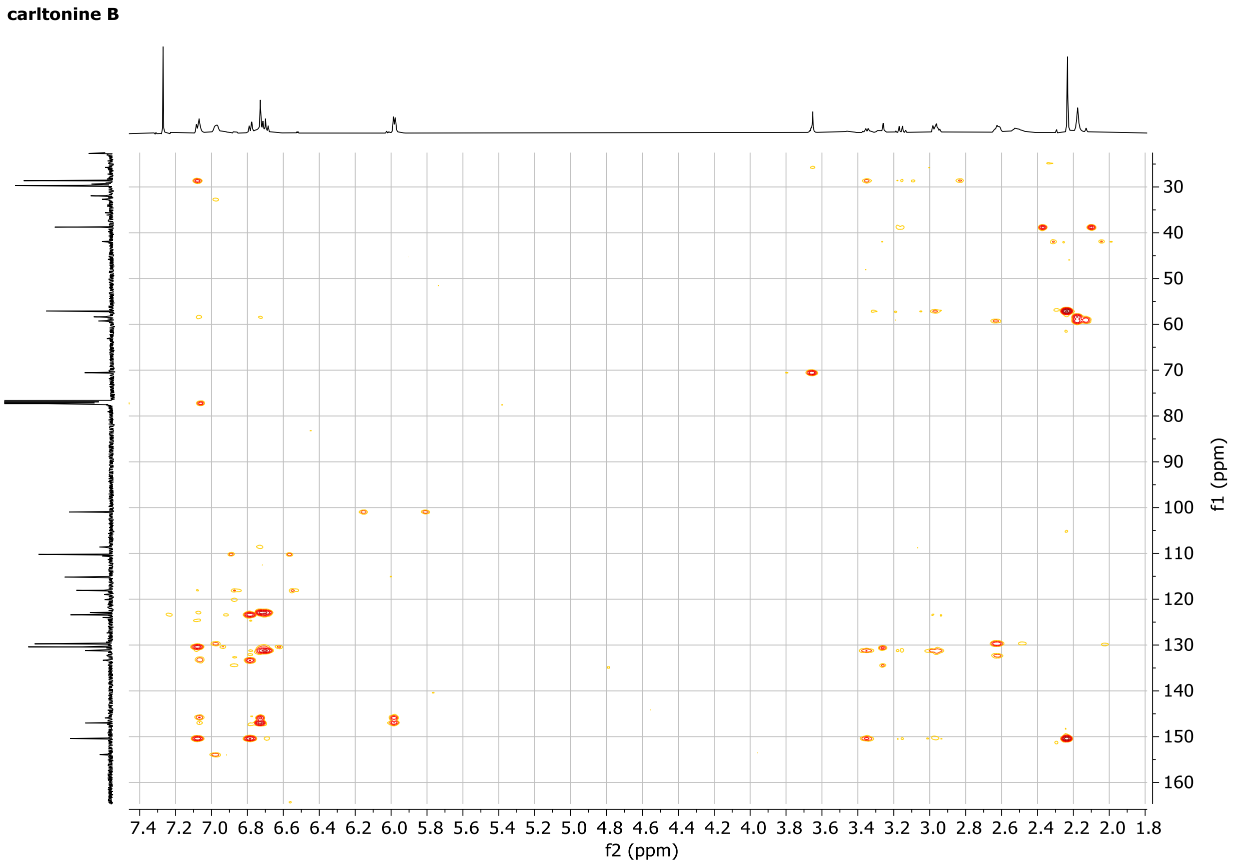

Figure S2-6. gHMBCAD spectrum of carltonine B (**14**) in $CDCl_3$.

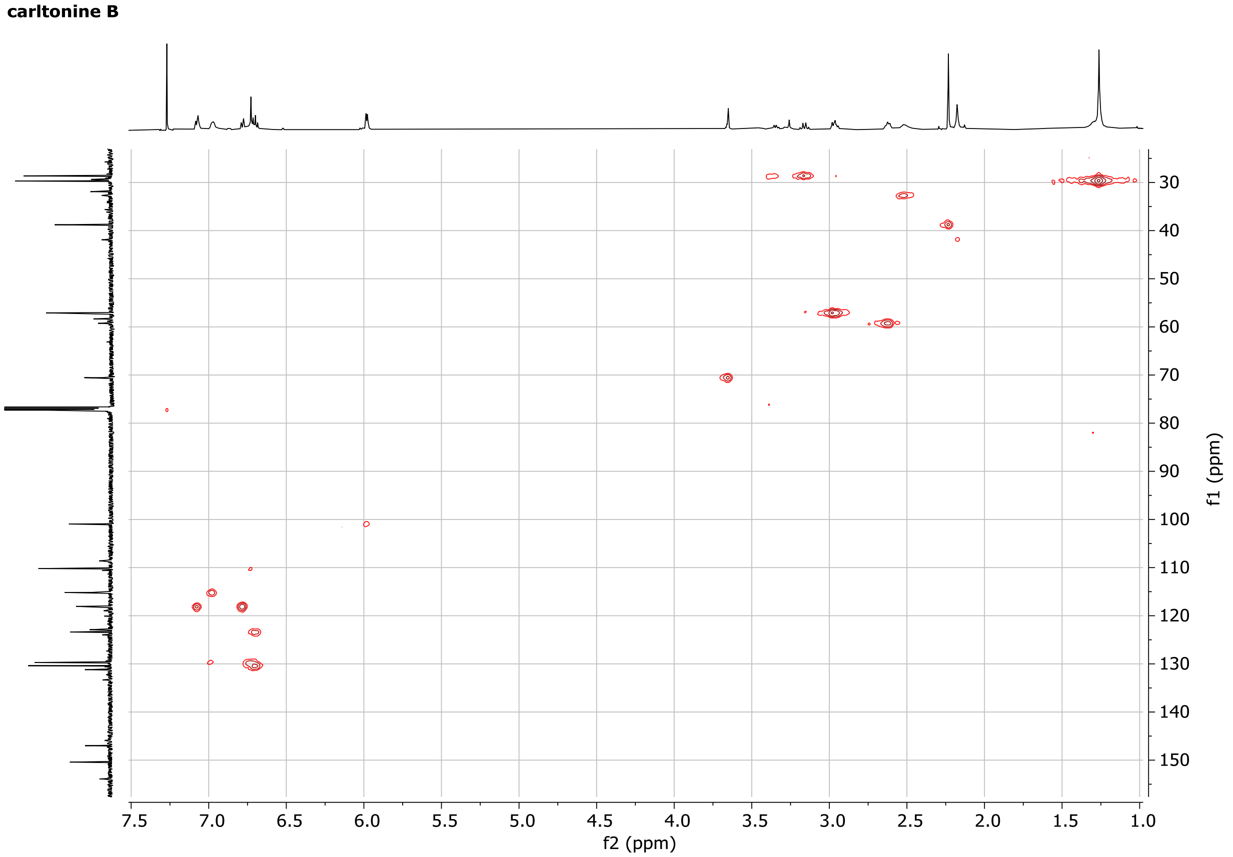

Figure S2-7. gH2BC spectrum of carltonine B (**14**) in $CDCl_3$.

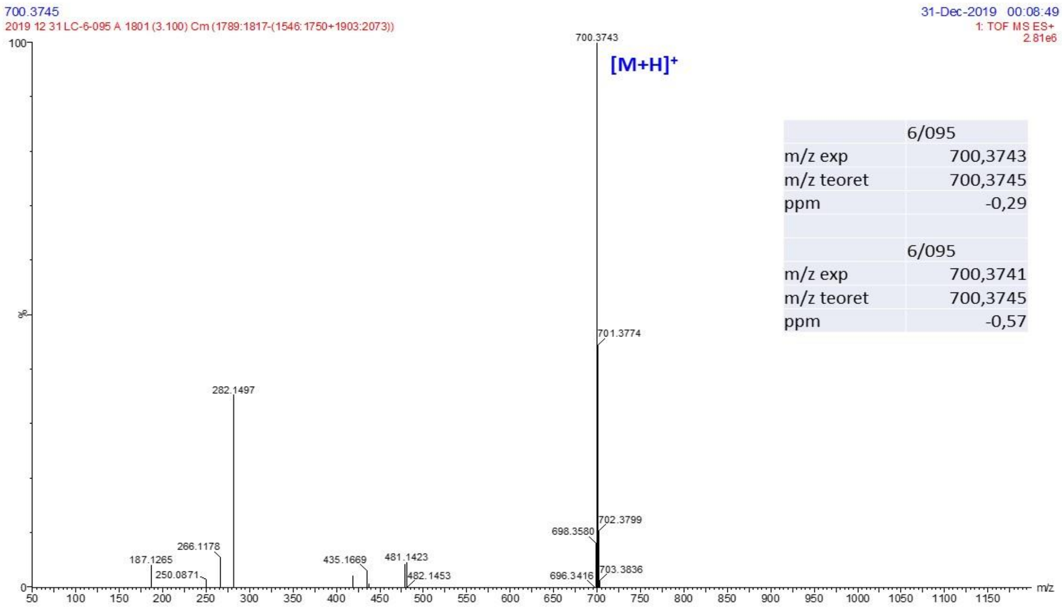

Figure S3-1. ESI-HRMS spectrum of carltonine C (15).

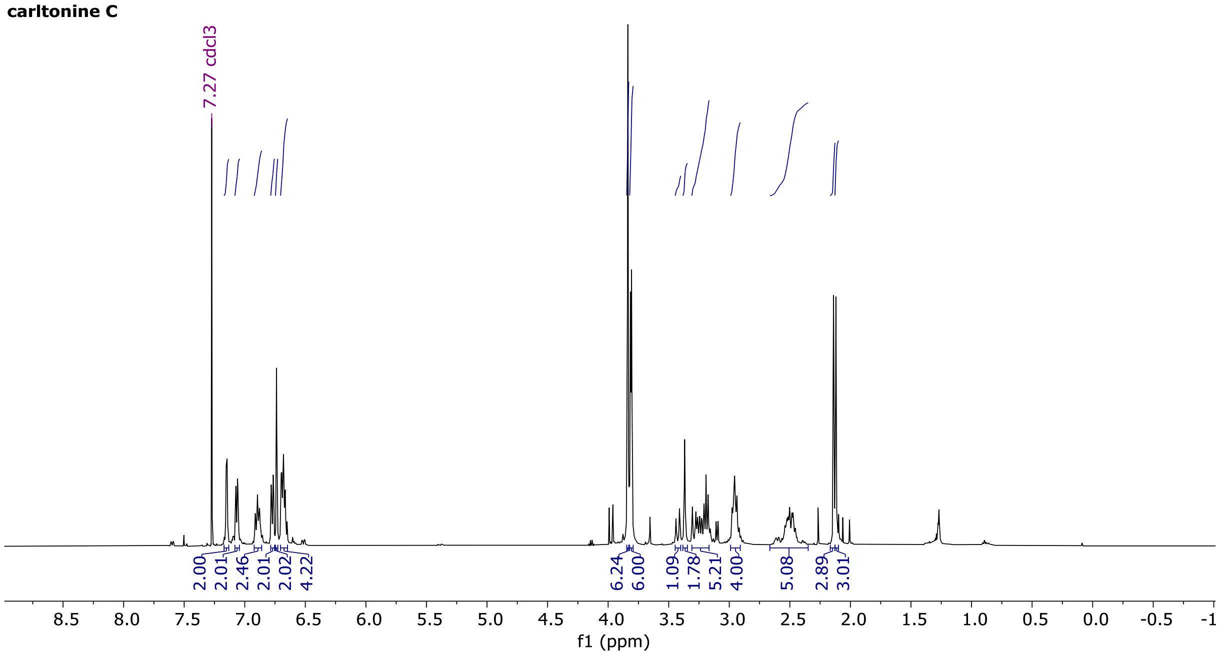

Figure S3-2. ^{1}H NMR spectrum of carltonine C (**15**) in $CDCl_3$.

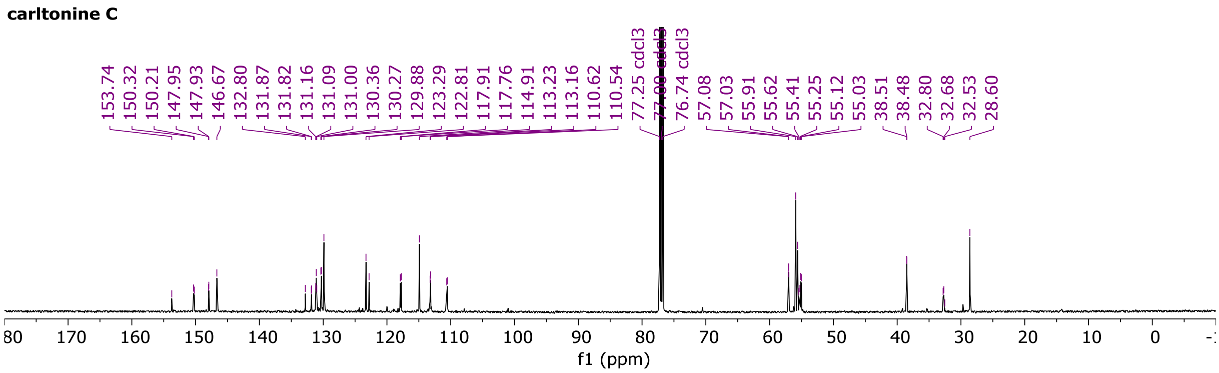

Figure S3-3. ^{13}C NMR spectrum of carltonine C (**15**) in $CDCl_3$.

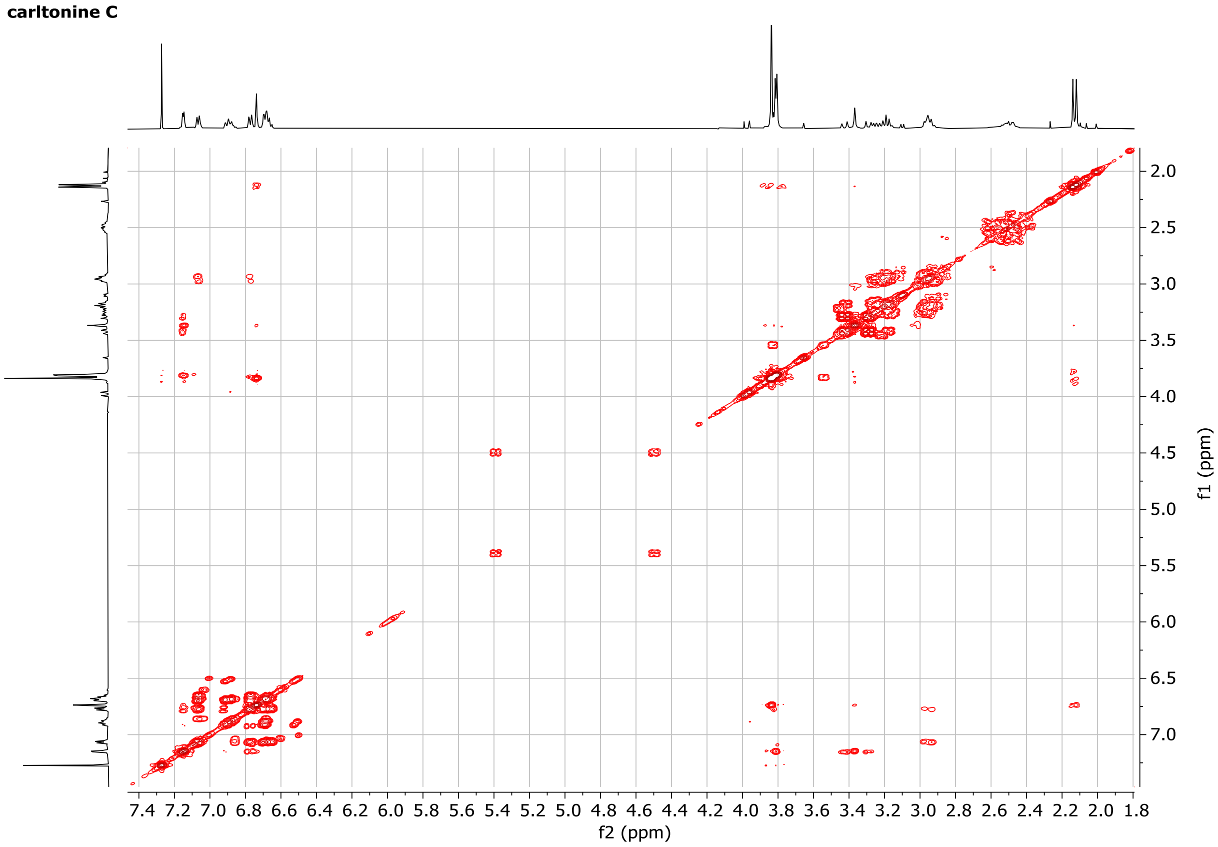

Figure S3-4. gCOSY spectrum of carltonine C (**15**) in $CDCl_3$.

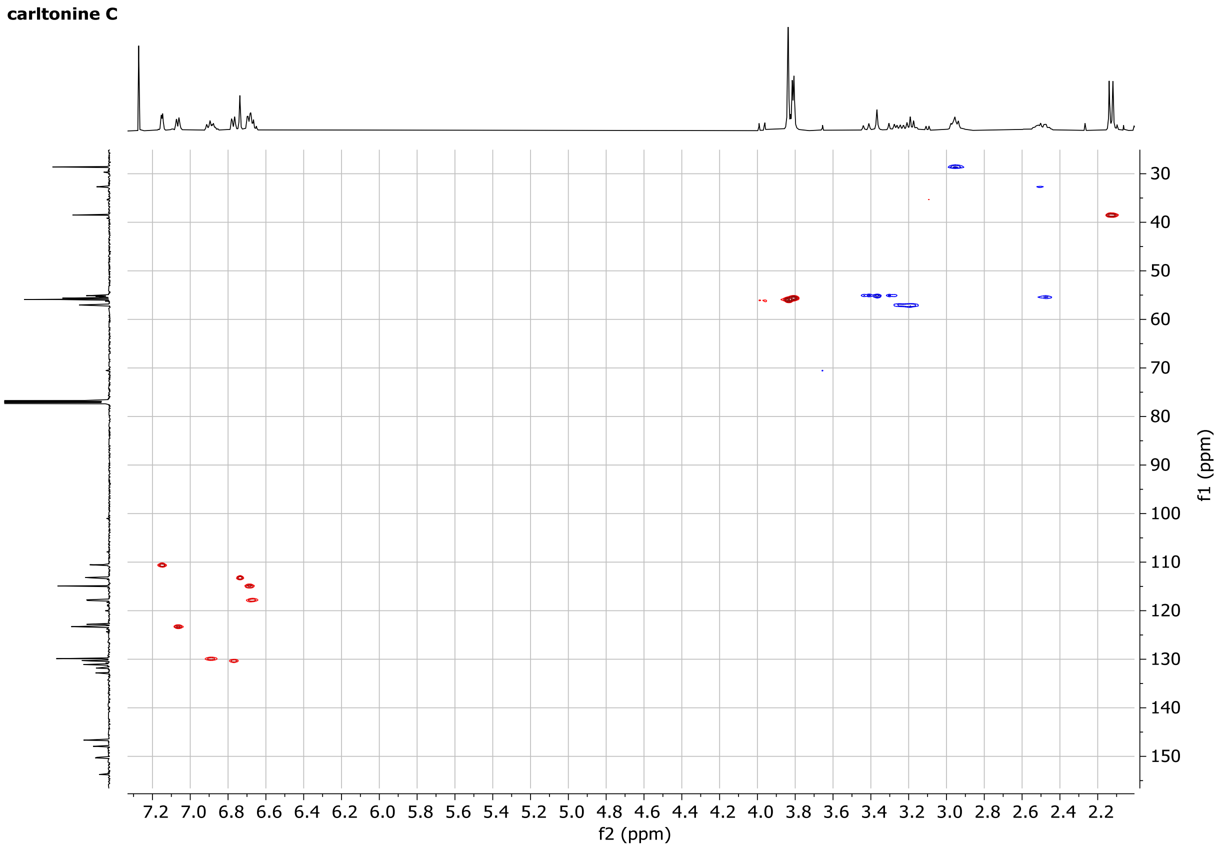

Figure S3-5. gHSQC spectrum of carltonine C (**15**) in $CDCl_3$.

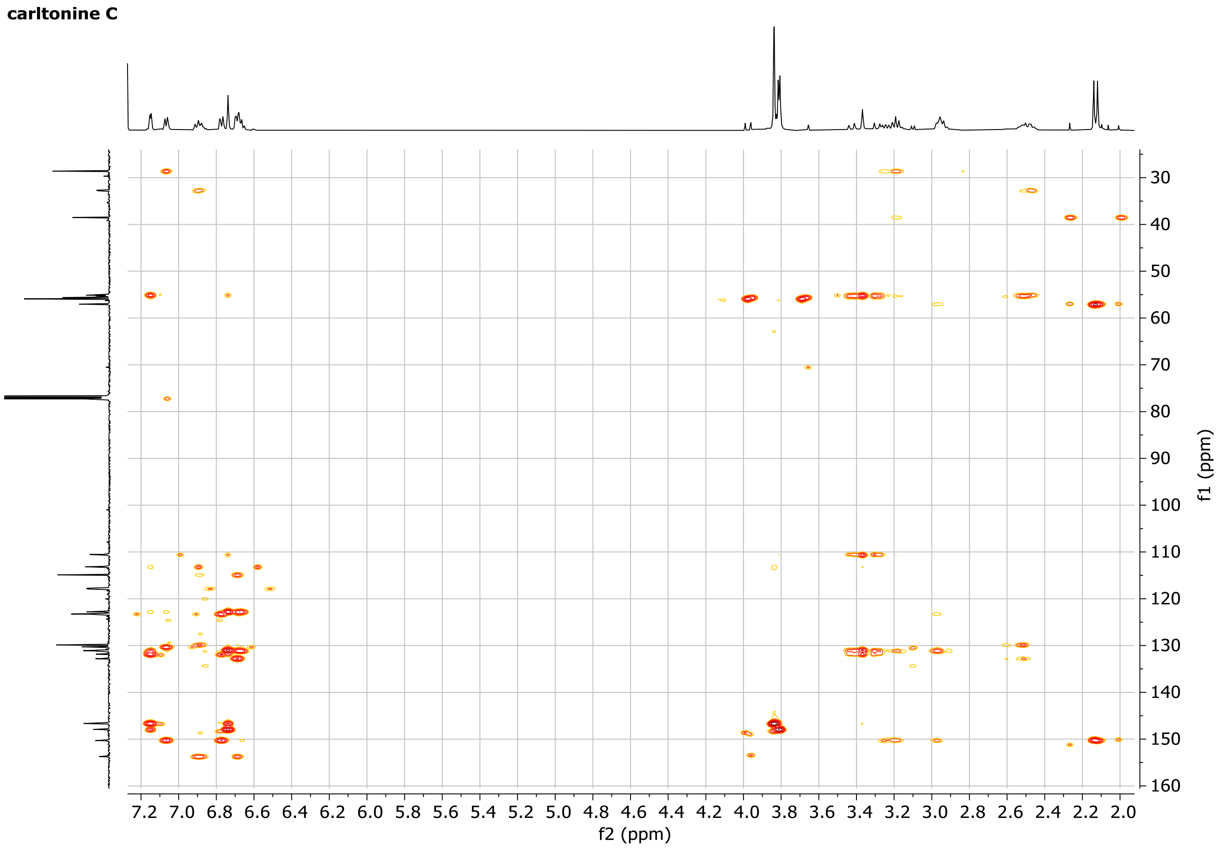

Figure S3-6. gHMBCAD spectrum of carltonine C (**15**) in $CDCl_3$.

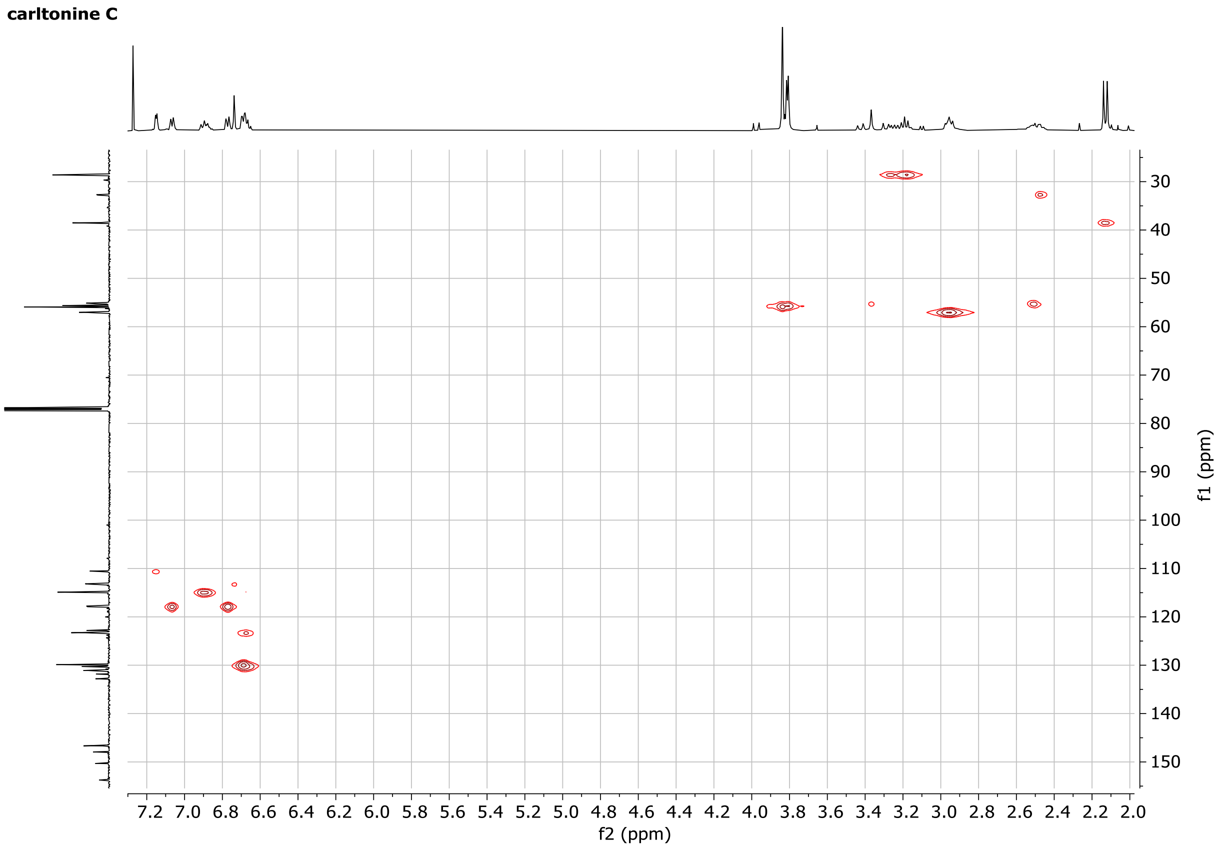

Figure S3-7. gH2BC spectrum of carltonine C (**15**) in $CDCl_3$.

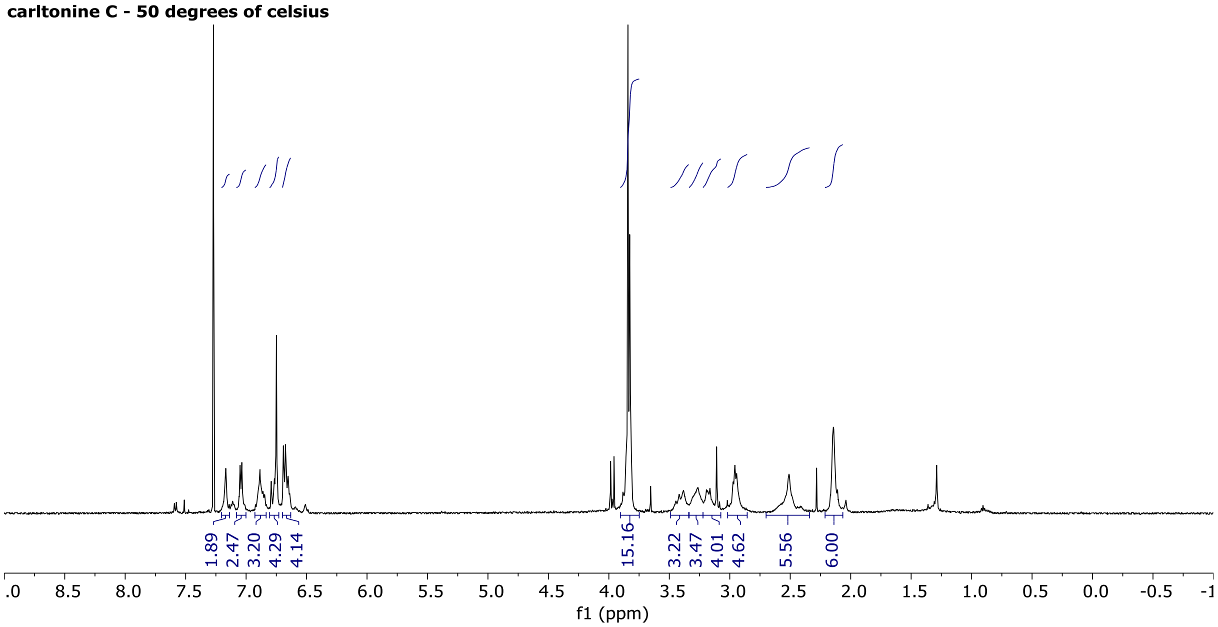

Figure S3-8. ^{1}H NMR spectrum of carltonine C (**15**) in $CDCl_3$ at 50 °C.

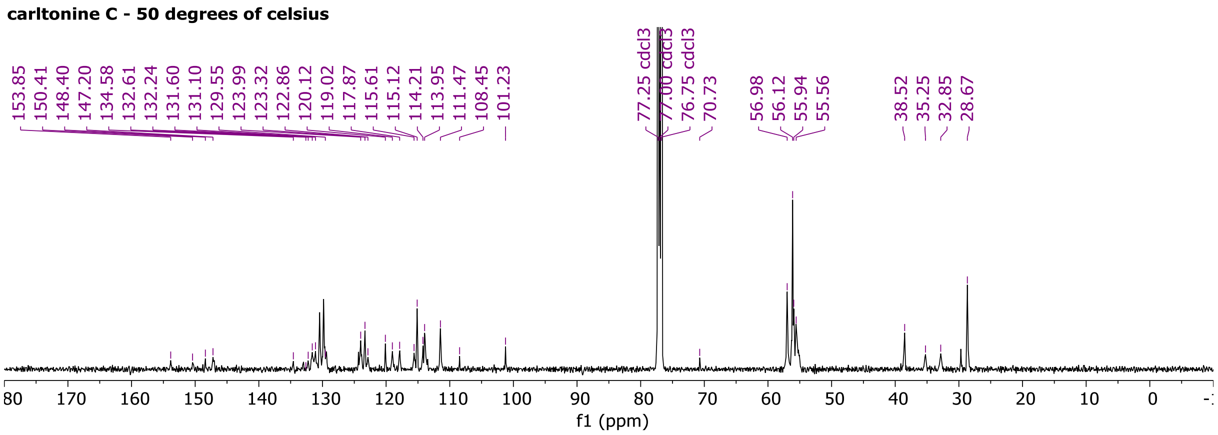

Figure S3-9. ^{13}C NMR spectrum of carltonine C (**15**) in $CDCl_3$ at 50 °C.

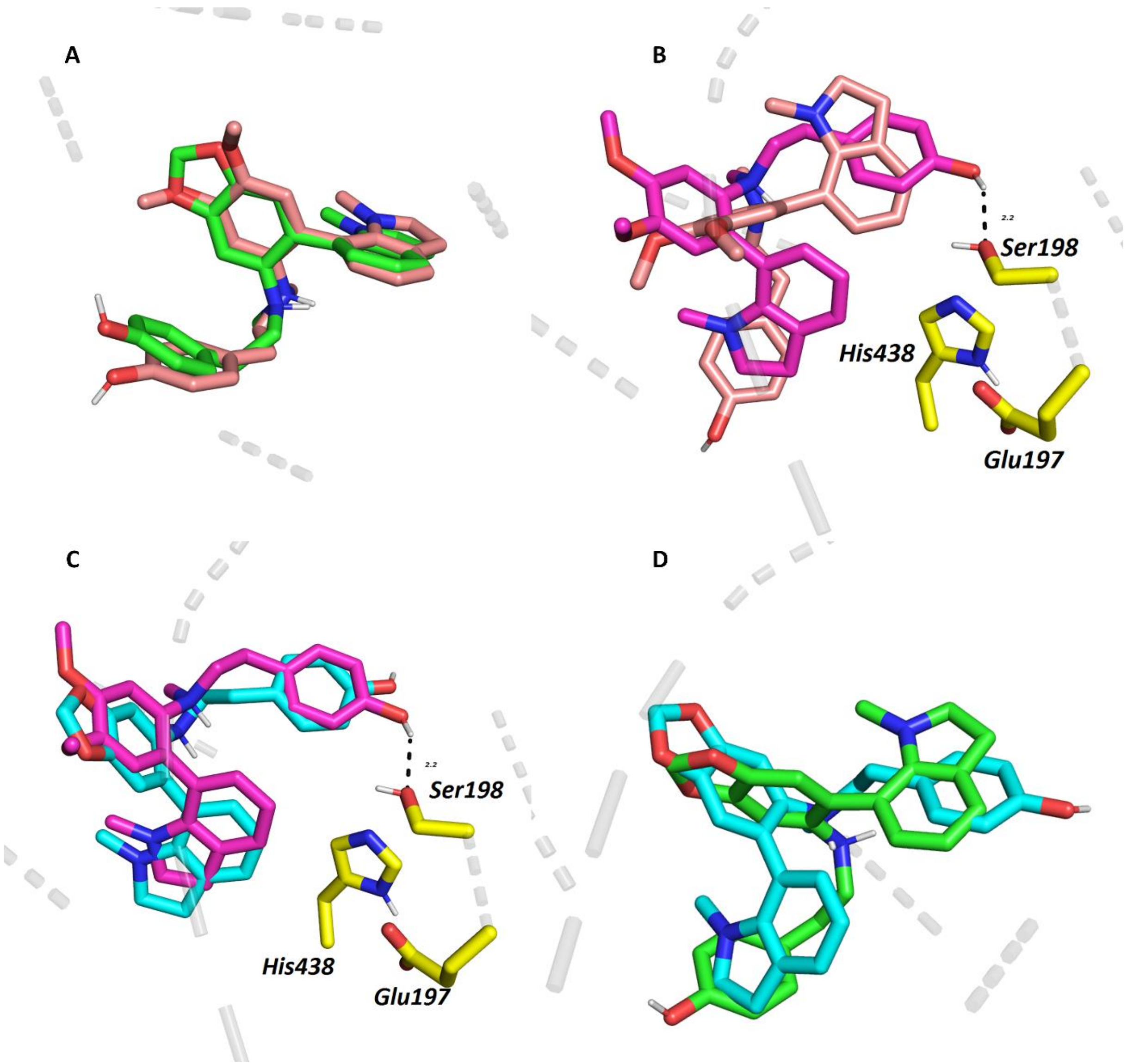

Figure S4. Overlapped *pseudo*-enantiomers in the *h*BuChE active site and their topology difference: (*R*)-**13**-(*R*)-**14** (A), (*S*)-**13**-(*R*)-**13** (B), (*S*)-**13**-(*S*)-**14** (C), and (S)-**14**-(*R*)-**14** (D). (*R*)-**13**, (*S*)-**13**, (*R*)-**14**, and (*S*)-**14** are shown in salmon, purple, green, and light blue, respectively. Catalytic triad residues are portrayed in yellow.
